# Estimated unit costs of anaemia interventions for women of reproductive age in 193 UN member states: a costing study

**DOI:** 10.1016/S2352-3026(25)00171-1

**Published:** 2025-08-26

**Authors:** Victoria L Oliver, Yingying Wang, Sumie Leung, Robin Blythe, Clare Glover-Wright, Jacinta Holloway-Brown, Michael Bode, Sant-Rayn Pasricha, Natalie Carvalho

**Affiliations:** a Melbourne School of Population and Global Health, University of Melbourne, Parkville, VIC, Australia; b Walter and Eliza Hall Institute of Medical Research, Parkville, VIC, Australia; c Duke-NUS Medical School, Singapore; d School of Mathematical Sciences, Queensland University of Technology, Brisbane, QLD, Australia; e School of Computer and Mathematical Sciences, University of Adelaide, Adelaide, SA, Australia; f Department of Clinical Haematology, Peter MacCallum Cancer Centre and The Royal Melbourne Hospital, Parkville, VIC, Australia

## Abstract

**Background:**

Anaemia affects an estimated 1·92 billion people worldwide. The UN Sustainable Development Goals set targets for reducing anaemia prevalence by 50% in women of reproductive age, for whom the risks and consequences of anaemia are the greatest. Prioritisation of cost-effective anaemia reduction strategies relies on robust estimates of the costs of interventions. We aimed to develop country-specific unit cost estimates for WHO-recommended anaemia interventions for women of reproductive age.

**Methods:**

A micro-costing approach was used to estimate unit costs (per recipient per year) for six anaemia prevention and treatment interventions in 193 UN member states using data from secondary sources. The interventions included were oral iron supplementation for pregnant and non-pregnant women, fortification of staple foods, multiple micronutrient supplementation for pregnant women, intermittent preventive treatment of malaria in pregnancy with sulfadoxine-pyrimethamine, presumptive deworming treatment for pregnant and non-pregnant women, and insecticide-treated bednets. A health-care sector perspective and 1-year timeframe were adopted. Cost categories included commodity, supply chain, service delivery, and administrative programme costs. Parameter uncertainty was explored in deterministic sensitivity analyses. Costs are presented as population-weighted means and SDs in 2023 US dollars.

**Findings:**

In most countries, staple food fortification and deworming were the lowest cost interventions, with population-weighted average costs of less than US$1 per person per year in settings with the highest burden of anaemia (ranging from $0·27 [SD 0·25] for staple fortification in low-income-countries to $0·84 [0·21] for deworming in non-pregnant women in lower-middle-income countries). Multiple micronutrient supplements had the highest average unit costs in most countries, with unit costs ranging from $9·57 (SD 0·58) in low-income countries to $135·56 (SD 25·95) in high-income countries. Commodity and service delivery costs were the largest cost drivers, although this varied across interventions and settings.

**Interpretation:**

Our standardised methodology and dataset estimate country-level unit costs and describe cost drivers for WHO-recommended anaemia interventions. These findings can facilitate cost-effectiveness analyses of anaemia interventions for women of reproductive age and strengthen priority-setting processes.

**Funding:**

Gates Foundation.

## Introduction

Anaemia is a major public health issue affecting an estimated 1·92 billion people worldwide.^1^ The condition can have several causes, including nutritional deficiencies (most commonly iron deficiency), infections (including HIV, malaria, and other parasitic infections), chronic disease, inflammation, gynaecological and obstetric conditions, and genetic conditions affecting red blood cell function.^2^ Anaemia in pregnancy elevates the risk of adverse outcomes for both mother and child, including preterm labour, postpartum haemorrhage, low birthweight, and stillbirth.^3^

Halving the prevalence of anaemia in women of reproductive age by 2025 was a goal of the 2012 WHO Global Nutrition Targets and reaffirmed in the 2015 UN Sustainable Development Goals (SDGs). However, progress towards these goals has been minimal and no country is on track to meet this target according to the most recent data from the Global Burden of Diseases, Injuries, and Risk Factors Study (GBD).^4^ Progress towards reducing the burden of anaemia hinges on implementation of effective interventions. Understanding the costs of these interventions is crucial to prioritise investment in those that are cost-effective and support resource mobilisation and budget planning efforts.

We aimed to estimate the per-person unit costs of all interventions recommended by WHO for reducing anaemia in women of reproductive age in 193 UN member states. Unit costs, representing the marginal cost of reaching each person with an intervention, can be used by national, regional, and global policy makers, researchers, and funding organisations to support cost-effectiveness analyses that are comparable between country settings and interventions. This presents an opportunity to bolster an evidence base to inform rational investments in anaemia reduction strategies, which will be crucial for driving progress towards reducing the burden of anaemia.

**Research in context**

**Evidence before this study**

We searched PubMed using terms for economic evaluations (eg, “cost-effectiveness analysis” OR “cost-benefit analysis”) AND anaemia intervention terms (eg, “iron supplementation” OR “micronutrient supplementation” OR “bednets”) AND terms for women of reproductive age (eg, “pregnant” OR “antenatal”). Although we found a considerable body of evidence describing the costs of anaemia interventions for women of reproductive age, these studies mostly focused on a small number of interventions or country settings. Across the studies, there was considerable variation in the methodological approach to estimating costs, which restricted opportunities to compare costs and cost-effectiveness between interventions and settings and prioritise those representing the best value. We searched Google and the “Resources” or “Tools and toolkits” pages of international agencies with work focused on nutrition and anaemia (WHO, World Bank, Global Alliance for Improved Nutrition, and Food Fortification Initiative) to identify publicly available costing tools. A World Bank analysis reported unit costs for multiple anaemia interventions; however, the analysis was limited to a sample of 26 countries with the highest prevalence of anaemia, and interventions without high-quality evidence of direct impact on anaemia, such as deworming treatments or insecticide-treated bednets, were not included. Publicly available costing tools, such as the OneHealth tool and the Optima Nutrition Tool, rely on users to supply country-specific input data to estimate costs and must be run for one country at a time.

**Added value of this study**

This study provides estimates of the annual per-person unit costs of interventions recommended by WHO for reducing anaemia in women of reproductive age in 193 UN member states. By using a costing approach that is consistent across interventions, while drawing on country-specific data where available, the results of this analysis allow for comparability of unit costs between interventions and country settings. These findings can support multicountry economic evaluations or the evaluation and prioritisation of multiple interventions within a single-country setting.

**Implications of all the available evidence**

These findings can be used by national, regional, and global policy makers, researchers, and funding organisations to support cost-effectiveness and budget impact evaluations of anaemia interventions that are comparable between country settings and interventions. This analysis presents an opportunity to bolster an evidence base to inform rational investments in anaemia reduction strategies.

## Methods

### Study design

Methods are reported in line with the Global Health Cost Consortium Reference Case for Estimating the Costs of Global Health Services and Interventions.^5^ An overview of the methods is presented below, with further detail on methods, assumptions, and data sources provided in the appendix (pp 1–21).

Calculations for estimating unit costs were done with Microsoft Excel (version 2502). To identify interventions, we reviewed the WHO e-Library of Evidence for Nutrition Actions (eLENA)^6^ and included those that are recommended by the WHO Guidelines Review Committee specifically for the prevention or treatment of anaemia or for improving iron status in women of reproductive age, referred to here as nutrition-specific interventions (table 1).^7^, ^8^, ^9^, ^10^, ^11^, ^12^, ^13^, ^14^, ^15^ These include oral iron supplementation for pregnant and non-pregnant women, fortification of staple foods, and multiple micronutrient supplementation (MMS) for pregnant women. We also included interventions cited by the WHO Guidelines Review Committee for their potential to improve anaemia status, albeit without high-quality evidence of their direct effect on anaemia prevalence, referred to here as nutrition-sensitive interventions. These include intermittent preventive treatment of malaria in pregnancy with sulfadoxine-pyrimethamine, presumptive deworming treatment for pregnant and non-pregnant women, and insecticide-treated bednets. Interventions that carry context-specific recommendations were costed only for country settings where those recommendations apply. Interventions that are not listed on the eLENA website were not included.

**Table 1:** Interventions, target populations, and recommended settings included in the analysis

	**Rationale for inclusion**	**Target population**	**Setting in which the intervention is recommended**
**Nutrition-specific interventions**			
Iron and folic acid supplementation	Explicitly recommended for the prevention of anaemia^7^, ^8^, ^9^	Menstruating women and pregnant women	Menstruating women: where anaemia prevalence is >20%; pregnant women: all settings
Fortification of rice, wheat flour, and maize or corn meal	Recommended to improve nutrient deficiency and iron status^10^, ^11^, ^12^	General population	In settings where staple foods are commonly consumed by the population; costs were estimated in settings where daily food availability is >75 g per person per day
Multiple micronutrient supplementation (MMS)	Recommended to improve nutrient deficiency in the context of rigorous research^7^, ^13^	Pregnant women	In the context of rigorous research; unit costs were estimated for all settings
**Nutrition-sensitive interventions**			
Intermittent preventive treatment of malaria in pregnancy with sulfadoxine-pyrimethamine	Reduction of anaemia cited in Guideline Evidence to Decision^14^	Pregnant women	In malaria-endemic settings; unit costs were estimated in settings where malaria prevalence is >1 per 1000 people
Insecticide-treated bednets	Recommended for implementation alongside intermittent preventive treatment of malaria during pregnancy with sulfadoxine-pyrimethamine^7^	General population	In malaria endemic settings; unit costs were estimated in settings where malaria prevalence is >1 per 1000 people
Deworming	Recommended to reduce the burden of helminth infections in settings with high prevalence of anaemia and evidence of reduction in severe anaemia cited in guidelines^7^, ^15^	Menstruating women and pregnant women	Where prevalence of hookworm or *Trichuris trichiura* infection, or both, is >20% and the prevalence of anaemia is >40%

Costs of interventions were estimated for 193 UN member states (appendix pp 6–10) using country-specific data from secondary sources where available (WHO, International Drug Price Indicator Guide, UNICEF supply catalogue, Global Fortification Data Exchange, and the International Labour Organisation). Where country-specific data were not available, we used a population-weighted average of data from equivalent WHO region and World Bank income classification subgroups.^16^ A formal health-care sector perspective was taken and both public and private sector costs were included for staple food fortification.^17^

Incremental costs, including start-up and recurrent costs, were calculated compared to a scenario without any coverage of the intervention. Unit costs represent the economic cost per recipient of the intervention per year, assuming best practice implementation of interventions at national scale. Costs were not discounted as a 1-year time horizon was taken. Costs associated with research, supporting change, or future costs were not included. Unless otherwise stated, an ingredients-based approach was taken, where the quantities (*q*) and prices (*p*) of inputs comprising each component were determined and final costs calculated as *q* × *p*.

### Resource use measurement

For all interventions except for staple food fortification, cost categories were defined in line with WHO Choosing Interventions that are Cost-Effective (WHO-CHOICE) methodology and included commodity costs (eg, cost of drugs and supplements); commodity supply chain costs; service delivery costs (eg, health facility visits); and programme costs, which capture the administrative level costs such as training, monitoring and evaluation, and community mobilisation.^18^

Commodity quantities were based on WHO-recommended dosing schedules for each intervention in each target group (appendix pp 1–2). The oral iron supplementation dosing schedule recommended by WHO varies depending on the national burden of anaemia, the woman's anaemia and pregnancy status, and her ability to tolerate daily iron as outlined in the appendix (p 5). In general, recommendations tend to promote higher doses for women with anaemia relative to those without (120 mg daily dosing compared with 30–60 mg); for pregnant women relative to non-pregnant women (daily dosing recommended unless it is not well tolerated), and for non-pregnant women in settings with a high prevalence of anaemia relative to lower prevalence settings (daily rather than weekly dosing is recommended where anaemia prevalence is >40%). Final resource quantity estimates for this intervention represent a weighted average of these variations in dosing schedules.

Interventions were assumed to be delivered through a combination of health facility visits, community health worker visits, and private pharmacies based on assumptions in the WHO OneHealth tool and the World Bank Investment Framework for Nutrition (appendix pp 13–16).^19^

### Pricing and valuation

Commodity prices in low-income and middle-income countries (LMICs) were taken from the International Medical Products Guide or the UNICEF price catalogue, with mark-ups applied to commodities purchased from private pharmacies (appendix p 11). In high-income countries, commodity prices were taken from a combination of listed private market prices and government procurement pricing databases, with no additional mark-ups applied. The costs of insecticide-treated bednets were taken from the UNICEF and Global Fund pricing catalogues and annualised assuming a 3% discount rate and 3-year lifetime.

Supply costs were estimated by inflating commodity costs by region-specific mark-ups published by WHO-CHOICE (appendix p 12). This supply chain mark-up was not applied to the commodities purchased from private retailers or to the commodity costs in high-income countries.

Health centre or hospital visits were valued with WHO-CHOICE outpatient visit unit costs.^20^ In many countries, community health workers are unsalaried; however, the economic cost of community health worker time was valued at country-specific minimum wage. For oral iron supplementation, the per-person cost of a point-of-care diagnostic test for anaemia was estimated to be US$0·50 and added to the service delivery cost.^21^ Sensitivity analyses used a lower estimate of 50% of the base case service delivery unit cost and an upper estimate of 150% of the base case. No service delivery costs have been applied to supplements purchased through private pharmacies as these costs are assumed to be reflected in the private sector mark-up applied to commodities accessed through these channels.

We estimated the costs of delivering insecticide-treated bednets through a combination of channels, including mass campaigns and health facility visits, drawn from a systematic review and meta-analysis.^22^ This cost was taken to encompass both service delivery and programme-level cost components (appendix p 17).

For all other interventions, programme costs were drawn from a study by Baltussen and colleagues,^23^ which modelled the start-up and ongoing costs of implementing a 10-year programme of oral iron supplementation in four WHO subregions (appendix p 17). This study adopted WHO-CHOICE methodology in the estimation of resource use and the associated costs, and assumed 95% coverage of the population. Programme unit costs for countries in regions not included in that analysis were drawn from the population-weighted average programme unit costs in countries with equivalent income classification.

Two case examples of how all inputs described above were used to estimate intervention unit costs are provided in the appendix (p 20).

Costs of wheat and maize flour fortification were estimated on the basis of data from a comprehensive study that estimated the total public and private sector costs associated with a national fortification programme in countries where introduction of fortification was deemed feasible based on assessment of consumption data and industrial structure.^24^ In our analysis, we assumed the programmes to be sufficient to supply fortified staple foods to 90% of the population in each country, and total costs were divided by the population in the year of the study to derive a unit cost per person (appendix pp 18–19). The lowest country-specific per-person cost was used as the lower estimate for all countries, while the highest country-specific per person cost was used as the upper estimate.

Estimates of rice fortification costs were drawn from a study in which private sector costs were reported as incremental costs to consumers per person per year in four countries.^25^ For the base case, we used the costs of fortification by cold extrusion in Costa Rica (US$0·55, 2008). The cost of fortification by coating in the USA (US$0·08, 2008) was taken as the lower estimate, and the cost of hot extrusion in China (US$1·68, 2008) was taken as the upper estimate.

Costs of staple food fortification have only been estimated for countries where daily food availability (a proxy for daily food intake per person) is greater than 75 g per person per day. Although a somewhat arbitrary threshold, this has been chosen as an indicator of where high rates of consumption might suggest that a major opportunity for fortification exists. This is a simplifying assumption, as country-level policy decisions on staple fortification are informed by several other considerations, such as industry structure and geographical and climatic conditions.^24^ Total per-person unit costs of fortification represented the average of the per-person cost of fortification of each staple food, weighted based on daily food availability.

### Adjusting for inflation and currency conversion

All costs were reported in 2023 US dollars, with adjustments for inflation and currency conversions made as outlined by Turner and colleagues.^26^ Briefly, costs of non-tradable goods (such as health worker salaries and programme costs) were first converted to local currency units (LCUs) with official exchange rates relevant to the year of data or purchasing power parity (PPP) conversion factor if source data were in international dollars. Costs in LCU were then adjusted for inflation to 2023 with GDP implicit price deflators, before finally converting to US dollars using 2023 exchange rates (or PPP conversion factor if source data were international dollars). Costs of tradable goods, such as drugs and insecticide-treated bednets, were first converted from LCUs to US dollars using exchange rates relevant for the year of data and then adjusted for inflation with the US inflation rate.

### Uncertainty analysis

Uncertainty in parameter inputs was explored in two sensitivity analyses. First, a one-way deterministic analysis was conducted to explore the impact of uncertainty in each cost component on the total per-person unit cost. In this analysis, costs were calculated using a lower or upper estimate for one cost component at a time while keeping the inputs for all other cost components at base case values. This analysis might represent an underestimate of the true uncertainty, since in real-world settings the value of cost components will not vary in isolation.^27^ The second analysis represented an extreme scenario analysis, where the maximum uncertainty associated with the unit cost of each intervention was explored by calculating unit costs using the lower or upper estimates for all cost components at the same time. This analysis will represent an overestimate of the true uncertainty as the values of cost components are not correlated in their variance.^27^

For both these analyses, upper and lower unit cost estimates were calculated for each country and aggregated by region or income group by use of population-weighted means. As such, aggregated results encompass country-to-country variability in unit costs as well as parameter uncertainty. A summary of parameter inputs that were varied in these sensitivity analyses is provided in the appendix (p 21).

### Role of the funding source

The study funder had no role in the study design, data collection, data analysis, data interpretation, or the writing of the report.

## Results

Base case intervention unit costs (per recipient per year), expressed as population-weighted means (and SDs) across income classifications and geographical regions are shown in table 2, with country-specific costs provided in the appendix (pp 22–28). In most countries, staple food fortification (rice, wheat or maize flour, or both) was the lowest cost intervention, followed by deworming tablets. Deworming tablets were the lowest cost intervention in six of the 12 UN member states for which unit costs were estimated, all of which were LMICs, primarily in Africa.

**Table 2:** Base case population-weighted average unit cost (per person) of anaemia interventions

		**Iron supplementation**		**IPTp-SP**	**Staple food fortification**	**Deworming**		**Insecticide-treated bednets**	**Multiple micronutrient supplementation**
		Pregnant women	Non-pregnant women	Pregnant women	All	Pregnant women	Non-pregnant women	All	Pregnant women
Income group*									
	High income	83·90 (26·06)	52·31 (14·74)	10·29 (2·50)†	0·12 (0·17)	NA	NA	8·48 (0·61)†	135·56 (25·95)
	Upper-middle income	10·37 (8·01)	6·95 (5·13)	3·49 (2·31)	0·67 (0·81)	NA	NA	8·71 (0·34)	16·62 (8·01)
	Lower-middle income	3·35 (3·73)	3·32 (3·49)	1·63 (0·41)	0·43 (0·22)	0·70 (0·19)	0·84 (0·21)	8·58 (0·99)	9·93 (3·73)
	Low income	2·63 (0·52)	2·53 (0·25)	1·41 (0·42)	0·27 (0·25)	0·46 (0·06)	0·65 (0·13)	6·31 (1·36)	9·57 (0·58)
WHO region									
	African region	3·30 (1·45)	2·94 (0·66)	1·65 (0·65)	0·51 (0·31)	0·47 (0·04)	0·62 (0·04)	7·83 (1·62)	10·39 (1·69)
	Region of the Americas	49·68 (50·91)	30·41 (30·56)	4·3 4 (2·69)	0·84 (1·34)	1·49 (0·00)‡	1·60 (0·00)‡	8·77 (0·69)	73·77 (72·58)
	Eastern Mediterranean region	13·02 (20·19)	9·60 (12·76)	1·46 (0·52)	0·10 (0·14)	0·59 (0·00)§	0·97 (0·00)§	6·16 (1·39)	22·98 (29·73)
	European region	44·71 (30·55)	23·30 (17·34)	NA	0·05 (0·05)	NA	NA	NA	77·14 (51·33)
	South-East Asian region	3·05 (0·90)	3·17 (0·38)	1·66 (0·32)	0·52 (0·16)	0·71 (0·00)¶	0·83 (0·00)¶	8·71 (0·49)	9·53 (0·86)
	Western Pacific Region	15·96 (22·90)	22·94 (21·37)	1·51 (0·59)	0·52 (0·10)	0·84 (0·12)	1·36 (0·19)	7·65 (1·74)	27·09 (36·85)
Global		18·20 (30·41)	14·51 (20·82)	1·91 (1·28)	0·46 (0·56)	0·62 (0·19)	0·78 (0·21)	8·20 (1·33)	31·59 (45·80)

Data are mean (SD), in US$ (2023). GNI=gross national income. IPTp-SP=intermittent preventive treatment of malaria in pregnancy with sulfadoxine-pyrimethamine. NA=not applicable (unit costs have not been estimated for interventions in settings where current WHO guidelines do not make explicit recommendations).

* According to World Bank classification. Low income, GNI lower than $1145; lower-middle income, GNI between $1146 and $4515; upper-middle income, GNI between $4516 and $14 005; high income, GNI greater than $14 006.^7^

† Estimate reflects unit costs in Panama, the only high-income country where malaria is endemic (prevalence >1 per 1000 people).

‡ Estimate reflects unit costs in Honduras, the only country in the region of the Americas where WHO recommendations for deworming would apply based on epidemiological profile.

§ Estimate reflects unit costs in Somalia, the only country in the Eastern Mediterranean region where WHO recommendations for deworming would apply based on epidemiological profile.

¶ Estimate reflects unit costs in Bangladesh, the only country in the South-East Asian region where WHO recommendations for deworming would apply based on epidemiological profile.

MMS for pregnant women had the highest unit cost in most countries, driven largely by the costs of tablets. In six LMICs (across the WHO African region, region of the Americas, South-East Asia region, and Western Pacific region), insecticide-treated bednets exceeded MMS as the highest cost intervention.

**Figure 1:**
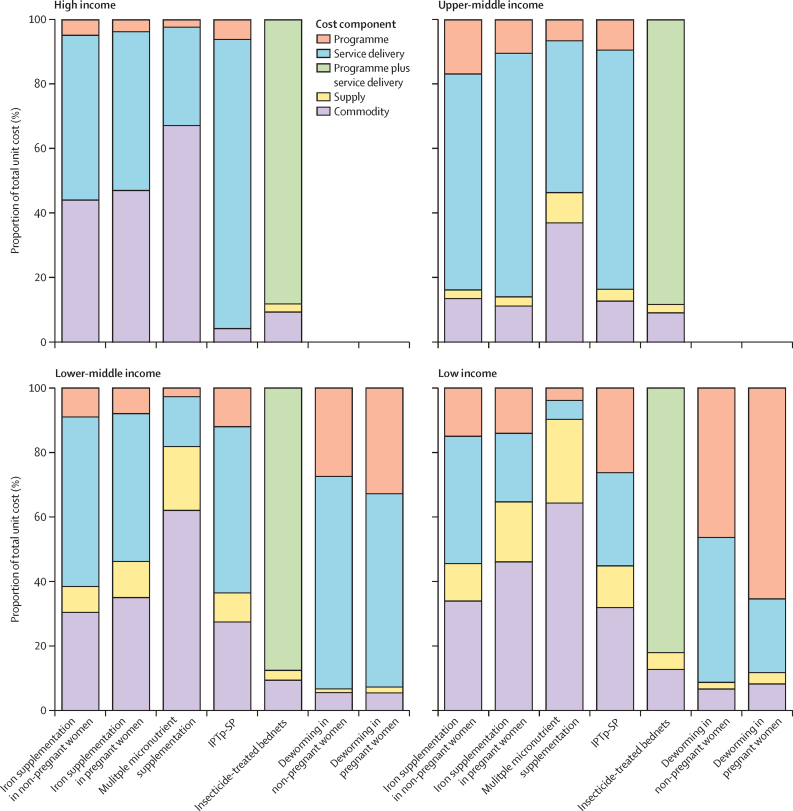
Breakdown of unit costs between cost components averaged across income group Unit costs of deworming interventions are not shown for high-income or upper-middle-income countries as they have only been estimated in countries where the prevalence of hookworm or *Trichuris trichiura* infection, or both, is greater than 20% and the prevalence of anaemia is greater than 40%, all of which are in low-income and lower-middle-income countries. Supply costs have not been included as a separate cost component in high-income countries as these costs have been assumed to be included in the private sector prices used to estimate commodity costs in these settings. IPTp-SP=intermittent preventive treatment of malaria in pregnancy with sulfadoxine-pyrimethamine.

The proportion of unit costs attributed to each component, averaged across income groups, is shown in figure 1, with averages across WHO regions shown in the appendix (p 29). Service delivery represented the largest component of the total unit costs of most interventions in high-income and middle-income settings except for MMS, for which commodity costs were the largest contributor to unit costs in most settings. These findings were similar for the WHO region of the Americas and South-East Asia region. Commodity or programme costs represented the largest share of most intervention unit costs in low-income countries and the WHO African region.

**Figure 2:**
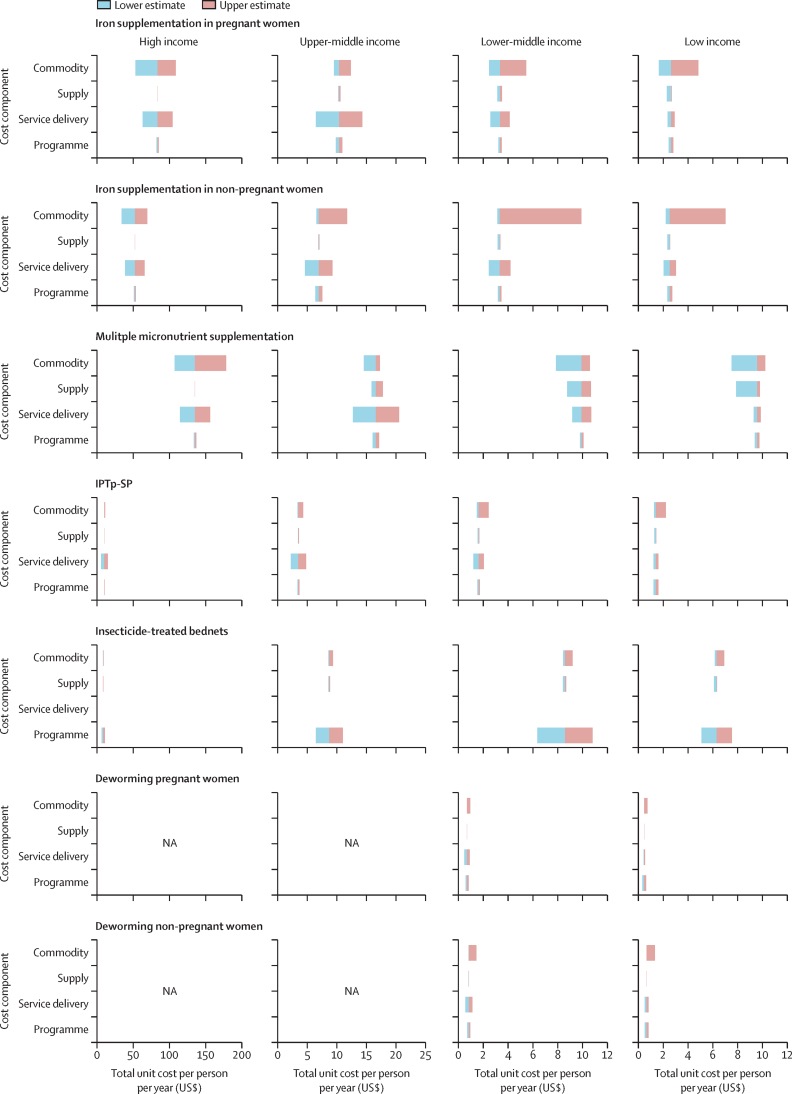
One-way sensitivity analysis of intervention unit costs averaged across income group Upper and lower bounds shown here represent population-weighted means of the upper and lower estimates from all countries in each income classification group. Only low-income and lower-middle-income countries had a high prevalence of hookworm or *Trichuris trichiura* infection, or both (>20%), and anaemia (>40%), hence unit cost estimates are not presented for high-income and upper-middle-income countries. Supply costs are not shown for deworming or IPTp-SP in high-income countries since these costs have been assumed to be included in the private sector prices used to estimate commodity costs in these settings. Programme costs for insecticide-treated bednets represent the combined cost of service delivery and programme costs; service delivery costs are not shown. IPTp-SP=intermittent preventive treatment of malaria in pregnancy with sulfadoxine-pyrimethamine. NA=not applicable.

Figure 2 shows the results from the one-way sensitivity analysis, averaged across income classification. In high-income countries, total unit costs were most sensitive to uncertainty in commodity costs for most interventions. In middle-income countries, unit costs for most interventions were most sensitive to uncertainty in commodity costs or service delivery costs. In low-income countries, unit costs were most sensitive to uncertainty in commodity costs for most interventions.

**Figure 3:**
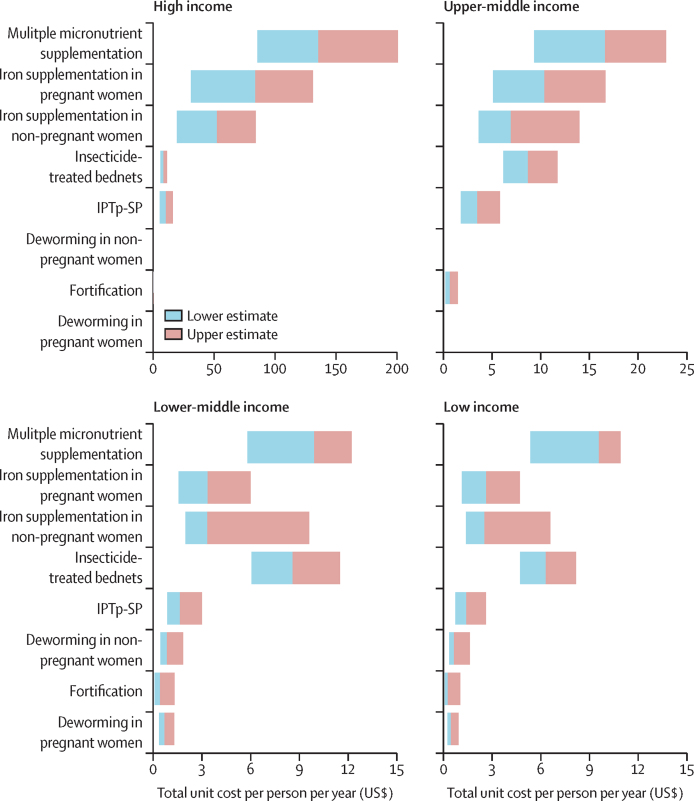
Extreme scenario analysis exploring total uncertainty in overall unit costs averaged across income group Upper and lower bounds shown here represent population-weighted means of the upper and lower estimates from all countries in each income classification group. Unit costs of deworming interventions are not shown for high-income or upper-middle-income countries as they have only been estimated in countries where the prevalence of hookworm or *Trichuris trichiura* infection, or both, is greater than 20% and the prevalence of anaemia is greater than 40%, all of which are in low-income and lower-middle-income countries. Fortification costs are shown for high-income countries, but the range (0·014–0·298) is not visible at this scale. IPTp-SP=intermittent preventive treatment of malaria in pregnancy with sulfadoxine-pyrimethamine.

Figure 3 shows the results from the extreme scenario analysis, averaged across income classification, with averages across WHO regions shown in the appendix (p 30). Across most income groups and regions, unit costs of MMS and oral iron supplementation interventions were associated with the greatest uncertainty. Unit costs of insecticide-treated bednets were also associated with considerable uncertainty in the WHO African and South-East Asian regions.

## Discussion

To the best of our knowledge, this is the first study to report country-specific cost estimates for WHO-recommended interventions for addressing anaemia in women of reproductive age. This work was conducted as part of *The Lancet Haematology* Commission on anaemia, informing novel approaches to setting targets for anaemia reduction over the next global development era.^2^ Our methodology provides consistent and comparable unit cost estimates for 193 countries, along with regional and income group summary estimates. Fortification of staple foods was generally the lowest cost intervention, with per-person unit costs estimated at less than US$1 per person per year in most countries where these costs were estimated. Among interventions specifically targeting women of reproductive age, preventive deworming therapy was the lowest cost intervention (average unit cost <$1).

Service delivery costs were often the largest cost component for interventions in high-income and middle-income countries, while commodity or programme costs were often the largest cost component in low-income countries. Estimates of the breakdown of unit costs by component could help inform governments and development partners on where to target strategies to minimise intervention costs. For example, in middle-income countries, identifying cost-efficient delivery channels might yield the largest impact in terms of reducing intervention costs. Similarly, effective pricing negotiations or market-shaping strategies, such as pooled procurement or priority review vouchers, will be important to drive down the cost of interventions for which commodity costs represent a large proportion of overall unit costs.

Sensitivity analyses were conducted to explore the impact of uncertainty in parameter inputs and assumptions made due to scarce availability of reliable cost data. These analyses showed that commodity costs and service delivery costs were the largest drivers of total unit costs, although there was some variation in the cost drivers between interventions and country settings. Future value of information analyses quantifying the expected returns from investing in reducing uncertainty would be beneficial to support policy decisions informed by these data.

Costs reported here represent economic costs and capture the full value of the inputs of each intervention (including, for example, donated goods or volunteered time). Economic costs are appropriate for this analysis, which primarily aims to generate data to support cost-effectiveness analyses.^5^ By contrast, financial costs represent the money that is spent on inputs and would support financial planning and budget impact analyses.^5^ Estimating financial costs requires knowledge of cost structures and procurement agreements, which vary considerably between settings, making them impractical to reliably determine for all countries included in this analysis.

These unit cost estimates represent the average marginal increase in the cost of reaching each additional person with the intervention per year. Unit costs represent the cost per recipient of the intervention, which might differ from the cost per person treated. For example, recipients of iron tablets who do not adhere to recommended dosing schedules when taking the tablets at home might not receive a therapeutic dose of the intervention. Reported rates of compliance with oral iron supplementation range from 72% to 92% for pregnant women, with rates in non-pregnant women likely to be even lower.^28^, ^29^, ^30^ In this study, we estimated costs on the basis of the assumption that iron tablets are provided to women along with counselling over several health-care visits, which might improve adherence to effective dosing regimens and thus the per-recipient unit costs reported here could be considered a reasonable approximation of the cost per person treated.

Programme startup costs have been included and as such unit costs are incremental compared to a scenario with no coverage of the intervention. In settings where an intervention has already been introduced and start-up costs already incurred, the unit cost reported here will overestimate the per-person cost of increasing coverage above baseline. However, programme startup costs have been distributed over a large population (95% coverage) and timeframe (10-year programme), and thus overestimation is considered minimal.

The inclusion of programme costs is a notable strength of this work, as activities that occur outside the health service delivery setting (eg, community sensitisation, monitoring, and evaluation) contribute to the full cost of an intervention but are not always included in costing studies or cost-effectiveness analyses.^31^ However, programme costs used in this analysis have been drawn from estimates developed by WHO-CHOICE in 2000.^31^ The methodology and data inputs used to estimate programme costs have since been updated to reflect the evolving complexity of these costs.^32^ However, these updated programme costs are not publicly available and so they could not be used for the present analysis.

The interventions included in this analysis were selected on the basis of those listed on the eLENA database and recommended by WHO in the context of addressing the burden of anaemia in women of reproductive age. This included three nutrition-specific interventions (oral iron supplementation; wheat flour, maize flour, or rice fortification; and MMS) and three anti-infectives (intermittent preventive treatment of malaria in pregnancy with sulfadoxine-pyrimethamine, insecticide-treated bednets, and deworming tablets). This list does not include other interventions, such as intravenous iron administration, pharmacotherapies addressing heavy menstrual or postpartum blood loss, and fortification of other foods (such as cassava, potatoes, sugar, and salt).^2^, ^33^ As the evidence base and global recommendations for anaemia reduction grow, future work to understand the costs and cost-effectiveness of these interventions is warranted.

Due to a paucity of country-specific data on the costs of staple food fortification, unit costs reported here rely on old data (studies conducted in 2008), and population-weighted averages of estimates from a small number of countries were used to estimate unit costs in countries where data were not available. These estimates should therefore be interpreted with caution, given that there is considerable variation between countries.^34^ We have also assumed a hypothetical coverage of 90% when using published estimates of total fortification costs to derive per-person costs. This will represent an underestimate of unit costs in settings where consumption of industrially processed staple foods is low, particularly if fixed costs represent a large share of the fortification costs. Country-specific data on the proportion of the population consuming industrially processed staples are available from the Global Fortification Data Exchange for two countries for maize flour, three countries for rice, and 13 countries for wheat flour. Coverage varies substantially from 10·6% for wheat flour in Uganda to 100% for rice in Costa Rica. More accurate estimation of the unit costs of staple food fortification would be facilitated by access to granular data on the fixed and variable costs of fortification, in addition to comprehensive country-specific data on the proportion of the population consuming industrially processed staple foods.

Unit costs are reported only from a health-care sector perspective. A societal perspective reflects the broadest public interest decision context and can include impacts on productivity, consumption unrelated to health, and educational achievement.^35^ However, few economic evaluations of anaemia and associated conditions have adopted a societal perspective,^36^ in part due to scarce robust evidence on the impact of anaemia on productivity and educational outcomes.^37^ Omission of a societal perspective from this analysis resulted in an underestimate of the economic benefits of interventions. However, this was considered reasonable due to the considerable uncertainty that would accompany estimates of costs and economic impacts beyond the health-care sector.

A further limitation of this analysis is that we did not explore cost heterogeneity to understand how unit costs vary in different locations or levels of coverage within a country, and it is likely that unit costs will vary non-linearly with intervention coverage. Although it is preferable to determine the cost function to describe this relationship between unit costs and scale, a substantial volume of data is required to do so, which are not widely available.

In summary, this work has provided estimates of the per-person, per-year unit costs of all interventions recommended by WHO for reducing anaemia in women of reproductive age in 193 UN member states. By using a common approach to costing, results of this analysis allow for comparability of unit costs between interventions and country settings. It is anticipated that these data can be used to support economic evaluations of anaemia interventions at national and global levels. These types of analyses will be important for identifying the most cost-effective anaemia interventions and for resource allocation planning to support efforts towards reducing the burden of anaemia worldwide. The accuracy and utility of the estimates we report could be strengthened with greater access to a range of data, underscoring the importance of the robust national surveys that underpin global health programmes.

### Contributors

### Data sharing

All input data used to estimate unit costs are provided in the Article and appendix. The MS Excel workbook used to calculate costs (https://doi.org/10.26188/28478285.v2), along with csv files presenting country-specific unit costs (https://doi.org/10.26188/28387226.v3 and https://doi.org/10.26188/28387172.v3) are publicly available on the University of Melbourne FigShare platform under the terms of the Creative Commons Attribution 4.0 International license (CC BY 4.0). This enables users to distribute, remix, adapt, and build upon the material in any medium or format, so long as attribution is given to the creator. These supporting data are accessible at https://doi.org/10.26188/c.7667258.v1. Any correspondence regarding the study or data used should be directed to the corresponding author.

## Declaration of interests

SRP has participated in advisory boards for CSL-Vifor, holds grants from the National Health and Medical Research Council, holds patents and receives royalties from Silence Therapeutics, and holds an unpaid role as Director of the WHO Collaborating Centre for Anaemia Detection and Control. Funding from the Gates Foundation was awarded to SRP and used to pay the salaries of NC, RB, VLO, SL, YW, and CGW (through payments to their institutions), and provide travel assistance for meetings and conferences for SRP, SL, MB, RB, NC, VLO, and YW. All other authors declare no competing interests.
