# Supplementary material for: Estimated unit costs of anaemia interventions for women of reproductive age in 193 UN member states: a costing study
Source: Lancet Haematol. 2025 Aug 26;12(9):e684–93. doi: 10.1016/S2352-3026(25)00171-1 (PMC12405068; doi:10.1016/S2352-3026(25)00171-1)
Supplement: Supplementary appendix [file mmc1.pdf]

THE LANCET
Haematology

Supplementary appendix

This appendix formed part of the original submission and has been peer reviewed. We post it as supplied by the authors.

Supplement to: Oliver VL, Wang Y, Leung S, et al. Estimated unit costs of anaemia interventions for women of reproductive age in 193 UN member states: a costing study. *Lancet Haematol* 2025; published online Aug 26. https://doi.org/10.1016/S2352-3026(25)00171-1.

1. Detailed Methods

1.1. Literature review

To identify interventions, we reviewed the WHO e-Library of Evidence for Nutrition Actions (eLENA) [1] and shortlisted those with WHO Guidelines available. Interventions were included if they were specifically recommended for the prevention or treatment of anaemia or for improving iron status in women of reproductive age. We also included interventions cited by the WHO Guidelines Review Committee in the "Evidence to Review" section for their potential to improve anaemia status, albeit with limited quality evidence of direct effect on anaemia prevalence. Interventions included are listed in Table A1, along with their associated recommendations.

Table A1: Interventions, target populations and dosing schedules included in the analysis.

Intervention	Target population	WHO recommendation
**Oral iron (& folic acid) supplementation**	Non-pregnant women of reproductive age	**Settings: < 20% prevalence of anaemia**<br>• No recommendations<br><br>**Settings: 20-40% prevalence of anaemia**<br>• Intermittent iron and folic acid supplementation are recommended with 60 mg of iron + 2800 mcg of folic acid once per week for 3 months on then 3 months off before supplementation again.<br>• If a woman is diagnosed as having anaemia in a clinical setting, she should be treated with daily iron (120 mg of elemental iron) and folic acid (400 µg or 0·4 mg) supplementation until her haemoglobin concentration rises to normal. She can then switch to an intermittent regimen to prevent recurrence of anaemia. [2]<br><br>**Settings: ≥40% prevalence of anaemia**<br>• Daily iron supplementation (30 – 60 mg) is recommended as a public health intervention in menstruating adult women and adolescent girls.<br>• If a menstruating woman is diagnosed with anaemia, national guidelines for the treatment of anaemia should be followed. [3]
	Pregnant women	**All settings**<br>• Daily oral iron and folic acid supplementation with 30 to 60 mg of elemental iron and 0·4 mg of folic acid is recommended for pregnant.<br>• If a woman is diagnosed with anaemia during pregnancy, her daily elemental iron should be increased to 120 mg until her Hb concentration rises to normal (Hb 110 g/L or higher). Thereafter, she can resume the standard daily antenatal iron dose to prevent recurrence of anaemia. [4]<br><br>**Settings: < 20% prevalence of anaemia**<br>• Intermittent oral iron and folic acid supplementation with 120 mg of elemental iron and 2800 mcg of folic acid once weekly is recommended if daily iron is not acceptable due to side-effects. [4]<br><br>**Settings: ≥40% prevalence of anaemia**<br>• A daily dose of 60 mg of elemental iron is preferred over a lower dose.
**Staple food fortification**	General population	Fortification of **rice** with iron is recommended as a public health strategy to improve the iron status of populations, in settings where rice is a staple food. [5]
		**Wheat flour** fortification should be considered when industrially produced flour is regularly consumed by large population groups in a country. [6]

		Fortification of **maize flour and corn meal** with iron is recommended to prevent iron deficiency in populations, particularly vulnerable groups such as children and women. [7]
**Intermittent preventive treatment with sulfadoxine-pyrimethamine (IPTp-SP)**	Pregnant women	IPTp-SP may reduce maternal anaemia (risk ratio: 0.90; 95% CI: 0.87–0.93; low-certainty evidence) and increase maternal haemoglobin (mean difference: 0.19 g/dL higher; 95% CI: 0.15–0.22 g/dL higher; low-certainty evidence) for each dose of SP in all gravidae. The effect is lower but remains significant in the highest SP resistance areas1 (relative risk reduction: 8.2%; 95% CI: 3–13%). IPTp-SP also reduced maternal anaemia in areas with PfPR2-10 < 3% (risk ratio: 0.91; 95% CI: 0.85–0.97). [8] In malaria-endemic areas in Africa, intermittent preventive treatment with sulfadoxine-pyrimethamine is recommended for all pregnant women. Dosing should start in the second trimester, and doses should be given at least one month apart, with the objective of ensuring that at least three doses are received. Each dose: Sulfadoxine (1500 mg)/Pyrimethamine (75 mg). [4]
**Insecticide treated bed nets**	General population	Universal access to and use of long-lasting insecticidal nets remains the goal for all people at risk of malaria. In endemic areas with intense malaria transmission (stable malaria), all infants at their first immunization and all pregnant women as early as possible in pregnancy should receive one long-lasting insecticidal net through immunization and antenatal care visits. [8]
**Deworming**	Women of reproductive age	Preventive chemotherapy (deworming), using annual or biannual single-dose albendazole (400 mg) or mebendazole (500 mg), is recommended as a public health intervention for all non-pregnant adolescent girls (10–19 years of age) and non-pregnant women of reproductive age (15–49 years of age) living in areas where the baseline prevalence of any soil-transmitted helminth infection is 20% or higher among non-pregnant adolescent girls and non-pregnant women of reproductive age. [9]
	Pregnant women	Preventive chemotherapy (deworming), using single-dose albendazole (400 mg) or mebendazole (500 mg) is recommended as a public health intervention for pregnant women, after the first trimester, living in areas where both: (1) the baseline prevalence of hookworm and/or *T. trichiura* infection is 20% or more and (2) where anaemia is a severe public health problem, with prevalence of 40% or higher among pregnant women. [4]
**Multiple Micronutrient Supplementation**	Pregnant women	Antenatal multiple micronutrient supplements that include iron and folic acid are recommended in the context of rigorous research. [10]<br><br>Limited dosing guidance available. Unit costs were estimated for a MMS dose equivalent to 30 mg elemental iron daily in line with other cost-effectiveness analyses. [11-13]

Interventions which carry context-specific recommendations were costed only for settings where those recommendations apply. Specifically, oral iron supplementation for non-pregnant women of reproductive age was not costed in setting where the prevalence of anaemia among women of reproductive age is less than 20%. Intermittent preventive treatment with sulfadoxine-pyrimethamine (IPTp-SP) and insecticide treated bed nets (ITN) were only costed in settings where the prevalence of malaria is > 1 per 1,000 people. Deworming tablets were only costed for setting where the prevalence of hookworm and/or *T. trichiura* infection is > 20% and the prevalence of anaemia among women of reproductive age is > 40 %.

Interventions which are not listed on the eLENA website were not included. Following selection of intervention, the key words outlined in Table A2 were used to search PubMed (All Fields) for previous costing studies.

Table A2: PubMed Database Keyword Search Strategy

**Iron or multiple micronutrient supplementation**

(cost-effectiveness analysis OR cost-benefit analysis OR economic analysis OR economic evaluation)<br>**AND**<br>(iron supplementation OR folic acid supplementation OR micronutrient supplementation)<br>**AND**<br>(pregnancy OR antenatal OR pregnant OR women of reproductive age OR menstruating)
**Insecticide treated nets (ITNs)**
(cost-effectiveness analysis OR cost-benefit analysis OR economic analysis OR economic evaluation OR costing)<br>**AND**<br>(bed nets OR insecticide treated bed nets)<br>**AND**<br>(pregnancy OR antenatal OR pregnant OR women of reproductive age OR menstruating)
**Antenatal antimalarials**
(cost-effectiveness analysis OR cost-benefit analysis OR economic analysis OR economic evaluation OR costing)<br>**AND**<br>(sulfadoxine OR pyrimethamine OR antimalarial OR malaria)<br>**AND**<br>(pregnancy OR antenatal OR pregnant OR women of reproductive age OR menstruating)
**Deworming**
(cost-effectiveness analysis OR cost-benefit analysis OR economic analysis OR economic evaluation OR costing)<br>**AND**<br>(Anthelmintic OR deworming)<br>**AND**<br>(pregnancy OR antenatal OR pregnant OR women of reproductive age OR menstruating)
**Fortification**
(cost-effectiveness analysis OR cost-benefit analysis OR economic analysis OR economic evaluation OR costing)<br>**AND**<br>(fortification) AND (rice OR wheat OR maize OR corn OR staple food)<br>**AND**<br>(pregnancy OR antenatal OR pregnant OR women of reproductive age OR menstruating)

1.2. Perspective and cost categories

Section 1.2 through to 1.6 describe the methods and assumptions used to estimate unit costs for oral iron supplementation, intermittent preventative treatment of malaria in pregnancy with sulfadoxine-pyrimethamine (IPTp-SP), insecticide treated bed nets (ITN), multiple micronutrient supplementation (MMS), and deworming therapy. Methods and assumptions used to estimate fortification unit costs are outlined in Section 1.7.

A formal health care sector perspective was taken for this analysis as defined by the Second Panel on Cost-Effectiveness in Health and Medicine. [13] This includes costs borne by the health care payer (whether the payer is government, insurance providers or development partners) as well as patient out-of-pocket healthcare costs.

In alignment with the WHO Choosing Interventions that are Cost-Effective (CHOICE) program[14] and the Second Panel on Cost-Effectiveness in Health and Medicine[13] the following cost categories were included in the unit costs:

- Patient level costs - costs are those incurred at the point of delivery of the intervention to the target population, and include:
    - Commodity costs – cost of the product associated with the intervention (eg iron tablet or bed net)
    - Costs of commodity supply – costs of transportation of product from site of manufacture to point of use
    - Cost of health service delivery– direct cost associated with provision of the product and necessary counselling to target population (health worker time or outpatient visits costs).
- Program level costs – costs outside of direct service delivery costs that are required to run the health program, such as administration, monitoring and evaluation, supervision, legislation, training and law enforcement.

Costs not included in the analysis are:

- Non-medical expenses incurred at the household level (such as travel, meals, and childcare expenses associated with seeking health services),
- Loss of productivity (due to illness, care seeking or care-giving) The decision to exclude these costs aligns with the adoption of a healthcare sector perspective, which does not include wider societal impacts, such as those affecting household earnings. This will lead to an underestimate of the full costs or cost-savings of an intervention.
- "Cost-offsets" (future health system costs that are averted due the intervention). While these costs are not included in the WHO-CHOICE methodology, they are considered part of the formal sector perspective according to the Second Panel. However, there is insufficient evidence available to estimate the future economic cost of anaemia, particularly in low-resource settings. No published cost-effectiveness analyses of anaemia interventions were identified that included future health systems costs averted.

1.3. Commodity costs

An ingredients-based approach was taken to estimating unit costs of commodities by first estimating the quantity of each commodity required for anaemia prevention or treatment per person per year (e.g. number of tablets) and multiplying this by the per quantity price (e.g. per tablet price).

To estimate commodity quantities, dosing schedules for each intervention in each target group were taken from the relevant WHO guidelines as outlined in Table A1. For iron supplementation see Figure A1 for an outline of dosing schedules, which vary based on the prevalence of anaemia, and a woman's anaemia status, pregnancy status and her ability to tolerate daily iron supplementation.

Figure A1: Flow chart of World Health Organization recommendations for oral iron supplementation dosing in women of reproductive age.

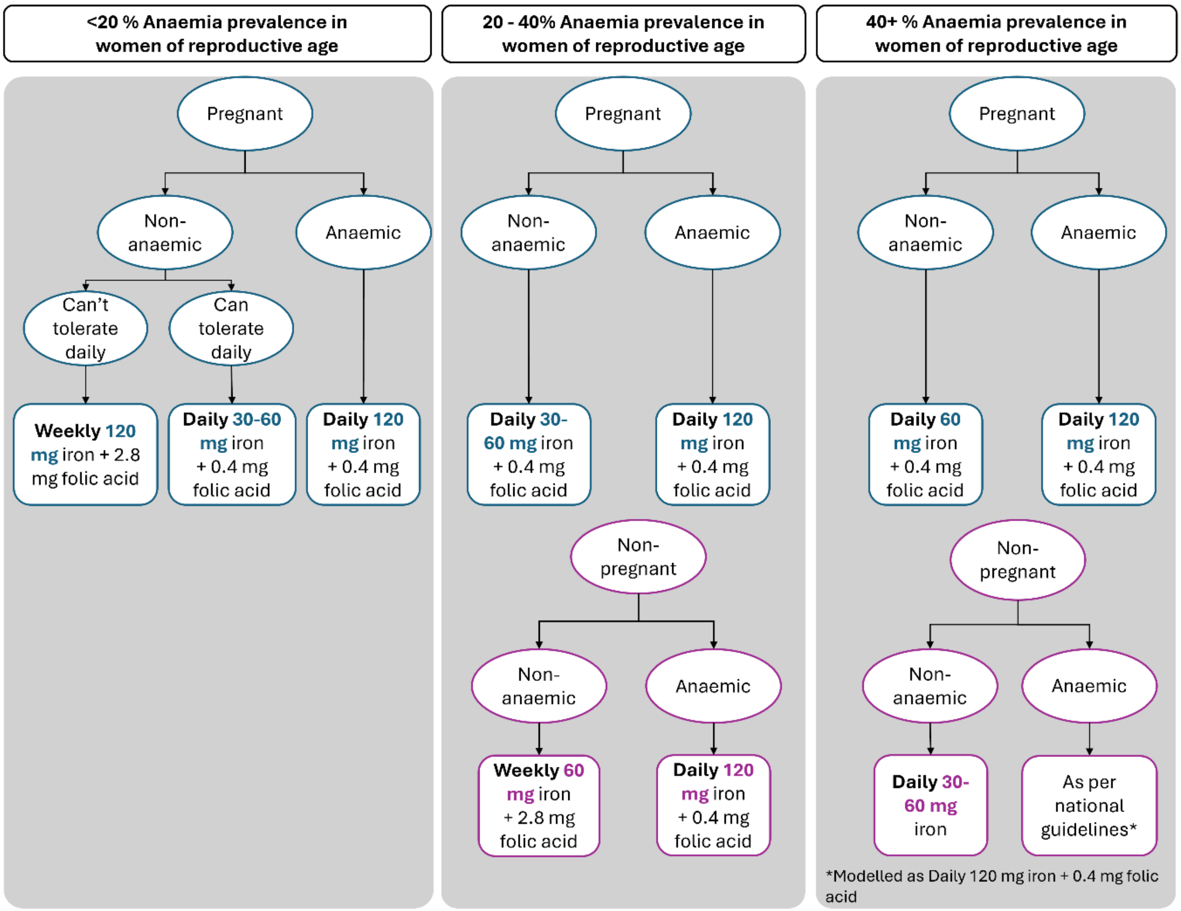

The WHO currently does not have any recommendations on the treatment of non-pregnant women in areas with low anaemia prevalence.

For iron supplementation, we assume a proportion of women are diagnosed with anaemia based on country-specific anaemia prevalence (see Table A3) and receive a treatment dose for 3 months before commencing on the

prevention dosing schedule. We assume 25% of pregnant women do not tolerate daily iron[15] and receive intermittent iron supplementation in settings and target groups where this dosing schedule is recommended. Final resource quantities represent a weighted average of the dosing schedules outlined in Figure A1.

Table A3: Country specific demographic, economic and epidemiological data.

UN Member State	WHO Region	Income classification[a]	Population ('000) 2021[b]	Prevalence of anaemia[c]	Coverage of ANC attendance	
					1+ visit[d]	*4+ visits[e]*
Afghanistan	EMR	Low-income	41,128·77	24·04%	65·2%	27·6%
Albania	EUR	Upper-middle-income	2,777·69	28·85%	88·4%	77·8%
Algeria	AFR	Lower-middle-income	44,903·23	36·06%	95·3%	69·8%
Andorra	EUR	High-income	79·82	7·96%		
Angola	AFR	Lower-middle-income	35,588·99	52·67%	81·6%	61·4%
Antigua and Barbuda	AMR	High-income	93·76	33·36%	100·0%	100·0%
Argentina	AMR	Upper-middle-income	46,234·83	17·61%	95·0%	90·4%
Armenia	EUR	Upper-middle-income	2,780·47	22·43%	99·6%	96·0%
Australia	WPR	High-income	26,014·40	8·86%	98·3%	92·0%
Austria	EUR	High-income	9,041·85	7·86%		
Azerbaijan	EUR	Upper-middle-income	10,141·76	35·58%	91·7%	66.1%
Bahamas	AMR	High-income	409·98	35·64%	98·0%	85·0%
Bahrain	EMR	High-income	1,472·23	42·71%	100·0%	100·0%
Bangladesh	SEAR	Lower-middle-income	171,186·37	44·42%	75·2%	36·9%
Barbados	AMR	High-income	281·64	30·33%	93·4%	87·9%
Belarus	EUR	Upper-middle-income	9,228·07	20·22%	99·9%	99·9%
Belgium	EUR	High-income	11,685·81	7·15%		
Belize	AMR	Upper-middle-income	405·27	40·51%	97·2%	92·6%
Benin	AFR	Lower-middle-income	13,352·86	66·71%	83·2%	52·1%
Bhutan	SEAR	Lower-middle-income	782·46	56·70%	97·9%	85·0%
Bolivia	AMR	Lower-middle-income	12,224·11	21·76%	95·6%	85·6%
Bosnia and Herzegovina	EUR	Upper-middle-income	3,233·53	23·50%	87·0%	84·2%
Botswana	AFR	Upper-middle-income	2,630·30	34·59%	94·1%	73·3%
Brazil	AMR	Upper-middle-income	215,313·50	34·16%	97·2%	92·2%
Brunei Darussalam	WPR	High-income	449·00	14·06%	99·0%	93·2%
Bulgaria	EUR	Upper-middle-income	6,465·10	25·52%		
Burkina Faso	AFR	Low-income	22,673·76	47·92%	98·3%	72·2%
Burundi	AFR	Low-income	12,889·58	38·34%	99·2%	49·3%
Cabo Verde	AFR	Lower-middle-income	593·15	42·21%	98·8%	85·6%
Cambodia	WPR	Lower-middle-income	16,767·84	41·79%	98·7%	86·3%
Cameroon	AFR	Lower-middle-income	27,914·54	37·64%	87·0%	64·9%
Canada	AMR	High-income	38,939·06	12·26%	100·0%	99·0%
Central African Republic	AFR	Low-income	5,579·14	38·36%	51·8%	41·4%
Chad	AFR	Low-income	17,723·32	59·14%	54·7%	31·0%
Chile	AMR	High-income	19,603·73	8·12%		
China	WPR	Upper-middle-income	1,412,175	15·82%	99·2%	92·9%
Colombia	AMR	Upper-middle-income	51,874·02	11·98%	97·2%	89·8%
Comoros	AFR	Lower-middle-income	836·77	38·41%	92·1%	48·9%
Congo	AFR	Lower-middle-income	5,970·42	48·31%	93·5%	79·0%
DR Congo	AFR	Low-income	99,010·21	29·38%	82·4%	42·9%
Costa Rica	AMR	Upper-middle-income	5,180·83	18·26%	97·6%	94·1%
Croatia	EUR	High-income	3,855·64	48·86%	98·4%	97·0%
Cuba	AMR	Upper-middle-income	11,212·19	19·31%	99·1%	79·3%
Cyprus	EUR	High-income	1,251·49	30·22%	99·2%	
Czechia	EUR	High-income	10,672·12	8·44%		
Cote d'Ivoire	AFR	Lower-middle-income	28,160·54	19·43%	95·2%	57·1%
Denmark	EUR	High-income	5,903·04	46·31%		
Djibouti	EMR	Lower-middle-income	1,120·85	8·26%	87·7%	22·6%

UN Member State	WHO Region	Income classification[a]	Population ('000) 2021[b]	Prevalence of anaemia[c]	Coverage of ANC attendance	
					1+ visit[d]	*4+ visits*[e]
Dominica	AMR	Upper-middle-income	72·74	37·32%	100·0%	
Dominican Republic	AMR	Upper-middle-income	11,228·82	33·95%	98·0%	92·6%
Ecuador	AMR	Upper-middle-income	18,001·00	33·57%	84·2%	57·5%
Egypt	EMR	Lower-middle-income	110,990·10	12·00%	90·3%	89·9%
El Salvador	AMR	Lower-middle-income	6,336·39	25·96%	96·0%	0·0%
Equatorial Guinea	AFR	Upper-middle-income	1,674·91	9·31%	91·3%	66·9%
Eritrea	AFR	Low-income	3,684·03	47·44%	88·5%	57·4%
Estonia	EUR	High-income	1,348·84	41·42%		96·8%
Eswatini	AFR	Lower-middle-income	1,201·67	18·86%	98·5%	76·1%
Ethiopia	AFR	Low-income	123,379·92	28·72%	73·6%	43·0%
Fiji	WPR	Upper-middle-income	929·77	23·10%	97·7%	88·7%
Finland	EUR	High-income	5,556·11	37·45%	99·8%	
France	EUR	High-income	67,971·31	7·29%	99·8%	98·9%
Gabon	AFR	Upper-middle-income	2,388·99	6·74%	94·7%	77·6%
Gambia	AFR	Low-income	2,705·99	57·03%	97·8%	78·5%
Georgia	EUR	Upper-middle-income	3,712·50	57·04%	97·6%	85·2%
Germany	EUR	High-income	83,797·99	25·23%	100·0%	99·3%
Ghana	AFR	Lower-middle-income	33,475·87	12·18%	97·4%	85·0%
Greece	EUR	High-income	10,426·92	58·10%		
Grenada	AMR	Upper-middle-income	125·44	10·09%	100·0%	
Guatemala	AMR	Upper-middle-income	17,357·89	32·70%	91·3%	86·2%
Guinea	AFR	Low-income	13,859·34	33·56%	86·0%	58·3%
Guinea-Bissau	AFR	Low-income	2,105·57	46·83%	97·0%	80·7%
Guyana	AMR	Upper-middle-income	808·73	56·47%	89·2%	84·7%
Haiti	AMR	Lower-middle-income	11,585·00	37·89%	91·0%	66·6%
Honduras	AMR	Lower-middle-income	10,432·86	58·80%	96·1%	88·3%
Hungary	EUR	High-income	9,643·05	16·34%		
Iceland	EUR	High-income	382·00	20·63%		
India	SEAR	Lower-middle-income	1,417,173·17	6·72%	85·1%	58·5%
Indonesia	SEAR	Lower-middle-income	275,501·34	55·66%	97·5%	77·4%
Iran	EMR	Lower-middle-income	88,550·57	28·14%	96·9%	94·3%
Iraq	EMR	Upper-middle-income	44,496·12	28·16%	87·6%	67·9%
Ireland	EUR	High-income	5,127·17	30·82%	99·5%	
Israel	EUR	High-income	9,557·50	7·61%		
Italy	EUR	High-income	58,940·43	9·69%	99·0%	68·1%
Jamaica	AMR	Upper-middle-income	2,827·38	8·45%	97·7%	85·6%
Japan	WPR	High-income	125,124·99	35·52%		
Jordan	EMR	Upper-middle-income	11,285·87	15·81%	97·6%	91·5%
Kazakhstan	EUR	Upper-middle-income	19,634·98	40·97%	99·3%	95·3%
Kenya	AFR	Lower-middle-income	54,027·49	38·73%	92·8%	58·5%
Kiribati	WPR	Lower-middle-income	131·23	24·11%	89·2%	0·0%
Kuwait	EMR	High-income	4,268·87	46·77%	100·0%	
Kyrgyzstan	EUR	Lower-middle-income	6,974·90	27·11%	100·0%	89·0%
Laos	WPR	Lower-middle-income	7,529·48	36·54%	78·4%	62·2%
Latvia	EUR	High-income	1,879·38	48·16%	91·8%	
Lebanon	EMR	Lower-middle-income	5,489·74	20·78%	95·6%	
Lesotho	AFR	Lower-middle-income	2,305·83	24·13%	91·3%	76·6%
Liberia	AFR	Low-income	5,302·68	28·66%	97·8%	87·3%
Libya	EMR	Upper-middle-income	6,812·34	71·94%	93·0%	
Lithuania	EUR	High-income	2,831·64	32·85%	100·0%	
Luxembourg	EUR	High-income	653·10	20·57%		97·3%
Madagascar	AFR	Low-income	29,611·71	7·62%	88·9%	59·9%
Malawi	AFR	Low-income	20,405·32	36·11%	96·8%	50·5%
Malaysia	WPR	Upper-middle-income	33,938·22	59·68%	99·4%	97·4%
Maldives	SEAR	Upper-middle-income	523·79	64·72%	98·7%	81·6%
Mali	AFR	Low-income	22,593·59	60·25%	84·2%	43·3%
Malta	EUR	High-income	531·11	59·64%	100·0%	
Marshall Islands	WPR	Upper-middle-income	41·57	9·16%	81·2%	77·1%

UN Member State	WHO Region	Income classification[a]	Population ('000) 2021[b]	Prevalence of anaemia[c]	Coverage of ANC attendance	
					1+ visit[d]	*4+ visits*[e]
Mauritania	AFR	Lower-middle-income	4,736·14	35·58%	85·0%	38·5%
Mauritius	AFR	Upper-middle-income	1,262·52	50·89%		
Mexico	AMR	Upper-middle-income	127,504·13	35·12%	98·5%	94·3%
Micronesia	WPR	Lower-middle-income	114·16	10·81%	80·0%	
Republic of Moldova	EUR	Upper-middle-income	2,538·89	40·44%	98·8%	95·4%
Monaco	EUR	High-income	36·47	6·45%		
Mongolia	WPR	Lower-middle-income	3,398·37	28·13%	99·1%	88·5%
Montenegro	EUR	Upper-middle-income	617·21	21·72%	97·2%	94·2%
Morocco	EMR	Lower-middle-income	37,457·97	34·86%	88·5%	53·5%
Mozambique	AFR	Low-income	32,969·52	57·28%	94·0%	51·0%
Myanmar	SEAR	Lower-middle-income	54,179·31	57·46%	80·7%	58·6%
Namibia	AFR	Upper-middle-income	2,567·01	30·30%	96·6%	62·5%
Nauru	WPR	High-income	12·67	42·68%	94·5%	40·2%
Nepal	SEAR	Lower-middle-income	30,547·58	50·53%	88·8%	77·8%
Netherlands	EUR	High-income	17,700·98	7·90%		
New Zealand	WPR	High-income	5,117·20	8·78%		
Nicaragua	AMR	Lower-middle-income	6,948·39	15·86%	94·6%	87·6%
Niger	AFR	Low-income	26,207·98	51·78%	93·8%	37·3%
Nigeria	AFR	Lower-middle-income	218,541·21	59·28%	69·6%	60·4%
North Korea	WPR	Low-income	26,069·42	26·86%	99·5%	93·7%
North Macedonia	EUR	Upper-middle-income	1,831·71	20·40%	97·3%	95·7%
Norway	EUR	High-income	5,457·13	6·93%		
Oman	EMR	High-income	4,576·30	32·97%	98·6%	73·9%
Pakistan	EMR	Lower-middle-income	235,824·86	56·43%	91·2%	52·4%
Palau	WPR	Upper-middle-income	18·06	37·18%	90·3%	81·0%
Panama	AMR	High-income	4,408·58	23·19%	99·1%	87·9%
Papua New Guinea	WPR	Lower-middle-income	10,142·62	53·84%	76·1%	49·0%
Paraguay	AMR	Upper-middle-income	6,780·74	29·32%	98·7%	85·1%
Peru	AMR	Upper-middle-income	34,049·59	26·60%	98·3%	95·8%
Philippines	WPR	Lower-middle-income	115,559·01	30·10%	93·8%	83·0%
Poland	EUR	High-income	36,821·75	22·84%		
Portugal	EUR	High-income	10,409·70	9·27%	100·0%	
Puerto Rico	AMR	High-income	3,220·11	25·19%		
Qatar	EMR	High-income	2,695·12	30·46%	90·8%	84·5%
Romania	EUR	High-income	19,047·01	24·77%	72·3%	76·0%
Russian Federation	EUR	Upper-middle-income	144,236·93	22·96%		
Rwanda	AFR	Low-income	13,776·70	21·77%	97·7%	47·2%
Samoa	WPR	Lower-middle-income	222·38	18·47%	94·1%	69·8%
San Marino	EUR	High-income	33·66	36·14%		
Saudi Arabia	EMR	High-income	36,408·82	37·65%	94·6%	79·7%
Senegal	AFR	Lower-middle-income	17,316·45	39·04%	97·6%	29·2%
Serbia	EUR	Upper-middle-income	6,664·45	27·22%	99·4%	96·6%
Seychelles	AFR	High-income	119·88	7·75%		
Sierra Leone	AFR	Low-income	8,605·72	42·78%	97·9%	78·8%
Singapore	WPR	High-income	5,637·02	33·00%		
Slovakia	EUR	High-income	5,431·75	67·65%	96·9%	
Slovenia	EUR	High-income	2,111·99	22·06%	99·5%	
Solomon Islands	WPR	Lower-middle-income	724·27	35·11%	88·5%	68·9%
Somalia	EMR	Low-income	17,597·51	43·78%	31·1%	24·4%
South Africa	AFR	Upper-middle-income	59,893·89	18·37%	93·7%	75·5%
South Korea	WPR	High-income	51,672·57	21·29%		96·8%
South Sudan	AFR	Low-income	10,913·16	17·61%	61·9%	31·1%
Spain	EUR	High-income	47,778·34	44·80%		
Sri Lanka	SEAR	Lower-middle-income	22,181·00	55·74%	98·8%	92·5%
St· Kitts and Nevis	AMR	High-income	47·66	34·81%	100·0%	
St· Lucia	AMR	Upper-middle-income	179·86	9·20%	96·9%	90·3%
St· Vincent and the Grenadines	AMR	Upper-middle-income	103·95	43·04%	99·5%	99·5%

UN Member State	WHO Region	Income classification[a]	Population ('000) 2021[b]	Prevalence of anaemia[c]	Coverage of ANC attendance	
					1+ visit[d]	*4+ visits*[e]
Sudan	EMR	Low-income	46,874·20	8·32%	79·1%	50·7%
Suriname	AMR	Upper-middle-income	618·04	36·31%	84·8%	67·5%
Sweden	EUR	High-income	10,486·94	44·45%	100·0%	
Switzerland	EUR	High-income	8,775·76	40·82%		
Syria	EMR	Low-income	22,125·25	7·60%	87·7%	63·7%
Sao Tome and Principe	AFR	Lower-middle-income	227·38	12·94%	98·1%	83·6%
Tajikistan	EUR	Lower-middle-income	9,952·79	35·30%	91·8%	64·2%
Tanzania	AFR	Lower-middle-income	65,497·75	41·16%	98·0%	62·2%
Thailand	SEAR	Upper-middle-income	71,697·03	23·17%	98·6%	90·0%
Timor-Leste	SEAR	Lower-middle-income	1,341·30	32·08%	84·4%	83·1%
Togo	AFR	Low-income	8,848·70	67·77%	77·9%	54·8%
Tonga	WPR	Upper-middle-income	106·86	35·92%	97·7%	88·5%
Trinidad and Tobago	AMR	High-income	1,531·04	36·48%	95·1%	100·0%
Tunisia	EMR	Lower-middle-income	12,356·12	26·79%	95·3%	84·1%
Turkey	EUR	Upper-middle-income	84,979·91	30·90%	96·4%	89·7%
Turkmenistan	EUR	Upper-middle-income	6,430·77	33·89%	100·0%	97·6%
Tuvalu	WPR	Upper-middle-income	11·31	41·65%	93·9%	60·3%
Uganda	AFR	Low-income	47,249·59	31·02%	95·1%	56·7%
Ukraine	EUR	Lower-middle-income	38,000·00	18·63%	98·6%	87·2%
United Arab Emirates	EMR	High-income	9,441·13	50·35%	100·0%	
United Kingdom	EUR	High-income	67,791·00	8·33%		
United States of America	AMR	High-income	333,271·41	49·98%		96·6%
Uruguay	AMR	High-income	3,422·79	12·27%	97·2%	96·8%
Uzbekistan	EUR	Lower-middle-income	35,648·10	12·53%	99·4%	89·5%
Vanuatu	WPR	Lower-middle-income	326·74	52·99%	75·6%	51·8%
Venezuela	AMR	Upper-middle-income	28,301·70	51·79%	97·5%	83·8%
Vietnam	WPR	Lower-middle-income	98,186·86	19·83%	97·0%	88·2%
Yemen	EMR	Low-income	33,696·61	24·95%	59·8%	25·1%
Zambia	AFR	Low-income	20,017·68	62·38%	96·9%	63·5%
Zimbabwe	AFR	Lower-middle-income	16,320·54	41·26%	93·3%	71·5%

AFR = African region; AMR = Region of the Americas; ANC = antenatal care; EMR = Eastern Mediterranean region; EUR = European region; WHO = World Health Organization

[a] According to World Bank classification: Low-income: GNI lower than $1,145; Lower-middle-income: GNI between $1,146 to $4,515; Upper-middle-income: GNI between $4,516 to $14,005; High-income: GNI greater than $14,006. [16]

[b] Source: United Nations Population Division. World Population Prospects: 2022 Revision.

[c] Prevalence or mild, moderate or severe anaemia in women of reproductive age (aged 15 – 49); Source: Global burden of disease estimates 2021.

[d] Percentage of women (age 15-19 and 15–49) attended at least once during pregnancy in the last or 5 years by skilled health personnel. Source: Most recent available data available at UNICEF Data Warehouse.

[e] Antenatal care 4+ visits - Percentage of women (age 15-19 and 15–49) attended at least four times during pregnancy by any provider. Source: Most recent available data available at UNICEF Data Warehouse.

Commodity prices were taken from a range of sources as outlined in Appendix Table A4. Briefly, for low- and middle-income countries (LMIC), commodity prices were taken from the International Medical Products Guide or the UNICEF price catalogue. Unless otherwise stated, the median estimate was used for the base case analysis, while the minimum and maximum reported prices were used as the lower and upper estimates for a sensitivity analysis respectively.

In LMIC settings, we assume that a proportion of the target population access supplements and pharmaceuticals through private retailers (see cost of health service delivery below for more detail) and apply an 83% mark up to

the commodity cost based on the average private sector mark-up on multiple micronutrient supplements reported by Bahl and colleagues. [17] A lower estimate of 10% was used in the sensitivity analysis based on the reported markup on pharmaceuticals in settings with highly regulated pharmaceutical pricing. [18] As an upper estimate we used the highest reported mark up in a setting with limited pricing regulations (171%).[19]

Table A4: Inputs for valuing commodity costs.

	Base case value (lower estimate, upper estimate), 2023 USD		Source
	Low- and-middle--income countries (LMIC)	*High-income countries (HIC)*	
Commodity prices			
Iron supplementation: 60 mg iron + 0·4 mg folic acid (daily dosing for anaemia prevention)	0·0131 (0·0045, 0·0251)	0·2247 (0·0502, 0·3898)	HIC: See note[a]. LMIC: See note[b]
Iron supplementation: 120 mg (daily dosing for anaemia treatment)	0·0075 (0·0055, 0·0276)	0·1998 (0·0493, 0·2247)	HIC: See note[a]. LMIC: See note[b]
Iron supplementation: 120 mg iron + 2·8 mg folic acid (intermittent dosing for anaemia prevention)[c]	0·0156 (0·0093, 0·0575)	0·3795 (0·0613, 0·5559)	HIC: See note[a]. LMIC: See note[b]
Sulfadoxine 500 mg; Pyrimethamine 25 mg (intermittent antimalarials)	0·0397 (0·0258, 0·1102)	0·0397 (0·0258, 0·1102)	See note[b]
Albendazole 400 mg/ Mebendazole 500 mg (deworming)	0·0383 (0·0354, 0·2877)	1·2827 (0·4167, 14·990)	HIC: See note[a]. LMIC: See note[b]
Insecticide treated bed nets	2·2800 (1·8700, 4·000)	2·2800 (1·8700, 4·000)	Both: Lower and base: UNICEF pricing catalogue; Upper: Global Fund pricing catalogue.
Multiple micronutrient supplementation	0·0337 (0·0224, 0·0372)	0·4982 (0·3453, 0·7330)	HIC: See note[a]. LMIC: UNICEF Supply catalogue
Mark-ups applied to commodities in private sector	83% (10%, 171%)	n.a.	See note[d]

HIC = high-income countries; LMIC = low- and-middle-income countries; n.a = Not applicable

[a] Three data sources were drawn on to estimate dose prices: UK pharmaceutical electronic market information tool (representing average price paid by public sector hospitals in the UK), prices listed by US-based private retailers (e.g. Walgreens), and estimated dispensed price estimated by the Australian Pharmaceutical Benefits Scheme. The median of the three sources was used as the base case, while the lowest and highest were used for the lower and upper estimates respectively.

[b] All estimates are from the International Medical Products Guide (adjusted for inflation using GDP-based deflators). Base case uses the median price, lower estimate uses the lowest listed price, upper estimate uses the highest listed price.

[c] The recommended dose of 120 mg iron + 2·8 mg folic acid is not currently on the WHO model essential medicines list and there are few manufacturers producing this formulation. [20] As such as dose price was based on 1 tablet of 5 mg folic acid and 2 tablets of 60 mg iron.

[d] Base case was based on the average private sector mark-up on MMS reported by Bahl and colleagues [17]. Lower estimate was based on the reported markup on pharmaceuticals in settings with highly regulated pharmaceutical pricing [18]. Upper estimate was based on the highest reported mark up in a setting with limited pricing regulations (171%) [19].

In high income countries, dose prices were taken from a combination of private market prices (Walgreens) and government procurement pricing databases (the UK government's Drugs and pharmaceutical electronic market information tool, and the Australian government's Pharmaceutical Benefits Scheme pricing database).

The costs of insecticide treated bed nets were taken from the UNICEF supply catalogue or Global Fund pricing catalogue and annualised assuming a 3% discount rate and 3-year lifetime.

1.4. Commodity supply costs

Commodity costs were inflated by region-specific mark-ups published by WHO-CHOICE to account for costs associated with transport of goods. Base case markups were used, which vary from 21% in EURO region to 44% in AFRO region (see appendix Table A5). This mark-up was not applied to the commodities accessed from private retailers, or to the commodity costs in high income countries as these costs were assumed to be already included in the commodity cost used.

In the sensitivity analysis, a lower estimate of 13% was used based on used in a recent WHO evaluation of the cost-effectiveness of a package of maternal, newborn and child health interventions. [21] The highest reported WHO-CHOICE markup (44% in the AFRO region) was used for all countries as the upper estimate.

Table A5: Commodity supply mark-ups used to estimate supply costs.

Region	Base case value, % (lower estimate, upper estimate)[a]	Source
AFR	44 (13, 44)	Base: WHO-CHOICE. Low: Stenberg, K., et al. [21] High: Highest WHO-CHOICE mark up
AMR	27 (13, 44)	Base: WHO-CHOICE. Low: Stenberg, K., et al. [21] High: Highest WHO-CHOICE mark up
EMR	33 (13, 44)	Base: WHO-CHOICE. Low: Stenberg, K., et al. [21] High: Highest WHO-CHOICE mark up
EUR	21 (13, 44)	Base: WHO-CHOICE. Low: Stenberg, K., et al. [21] High: Highest WHO-CHOICE mark up
SEAR	30 (13, 44)	Base: WHO-CHOICE. Low: Stenberg, K., et al. [21] High: Highest WHO-CHOICE mark up
WPR	23 (13, 44)	Base: WHO-CHOICE. Low: Stenberg, K., et al. [21] High: Highest WHO-CHOICE mark up

AFR = African region; AMR = Region of the Americas; EMR = Eastern Mediterranean region; EUR = European region; SEAR = South-East Asian region; WHO-CHOICE = World Health organization WHO Choosing Interventions that are Cost-Effective; WPR = Western Pacific Region

[a] Data represents % mark-up applied to commodities obtained outside of the private sector in low- and-middle-income countries only. Supply cost markups have been applied to the costs of ITN in all settings, as commodity costs were drawn from non-commercial suppliers (UNICEF and Global Fund) for all malaria endemic countries, regardless of-income classification.

1.5. Cost of health service delivery

An ingredients-based approach was taken to estimating unit costs of service delivery by first estimating the 'quantity' of each service delivery mechanism required for anaemia prevention or treatment per person per year (e.g. number of healthcare visits and proportion of visit dedicated to anaemia intervention) and multiplying this by the per quantity unit cost (e.g. cost of a healthcare visit).

1.5.1. Interventions targeting pregnant women

Provision of all interventions (except ITN) to pregnant women was assumed to take place through a combination of antenatal care (ANC) and community health worker visits as outlined in Table A6. For each intervention we quantify resource inputs as 2 minutes of an ANC visit (20% of the whole visit) or 5 minutes of a community health worker visit. We assume iron supplements are delivered through 4 visits (of either ANC or community health worker), antimalarials through 3 visits and deworming tablets through 1 visit. We drew on country-specific estimates of coverage of 4+ ANC visit to model the proportion of women who receive iron supplements and antimalarials through ANC visits. Country-specific estimates of coverage of at least 1 ANC visit were used to model the proportion of women who receive deworming tablets through ANC. The remainder of women were assumed to receive the interventions through community health workers and the final cost estimate represents a weighted average of these two delivery channels.

We have assumed that ANC is delivered through a combination of health service settings as outlined in Table A6. This distribution is based on data from demographic and health surveys on the percentage of women who report receiving ANC from a doctor (used as proxy for hospital setting) compared to another provider (used as a proxy for a health centre). We recognise considerable uncertainty in these estimates given that provider of ANC is only a weak indicator for setting for care. Care provided by a doctor may take place at a health centre and equally antenatal care at a hospital may be delivered by a nurse/midwife. Further there is likely to be considerable variation between countries within one income classification in the settings at which antenatal care is provided. However, reliable country-specific data on the settings of ANC are not widely available in the public domain.

Table A6: Inputs for estimating service delivery costs in pregnant women.

	Intervention		
	Iron supplementation	*IPTp-SP*	*Deworming*
Health facility visits			
% of women accessing intervention through this channel	Based on country specific coverage of 4+ ANC visits	Based on country specific coverage of 4+ ANC visits	Based on country specific coverage of 1+ ANC visits
% of ANC visits at hospitals in low-income countries[a]	20%	20%	20%
% of ANC visits at hospitals in lower-middle-income countries[a]	40%	40%	40%
% of ANC visits at hospitals in upper-middle-income countries[a]	65%	65%	65%
% of ANC visits at hospitals in high-income countries[a]	90%	90%	90%
Number of visits for delivery of intervention[b]	4	3	1
Proportion of visit dedicated to delivery of intervention[b]	20%	20%	20%
Community health worker visits			
% of women accessing intervention through this channel	Remaining women not accessing intervention through ANC	Remaining women not accessing intervention through ANC	Remaining women not accessing intervention through ANC
Number of visits for delivery of intervention[b]	2	3	1
Proportion of visit dedicated to delivery of intervention[b]	5 minutes	5 minutes	5 minutes

ANC = antenatal care; IPTp-SP = Intermittent preventative treatment of malaria in pregnancy with sulfadoxine-pyrimethamine.
[a] Proportion of women reporting that their provider of ANC was a 'doctor' has been used as proxy to guide estimation of the proportion of ANC visits that take place at a hospital with the remaining visits assumed to take place at a health centre (based on Demographic and Health Survey data compiled at STATCompiler). An ANC visit at a health centre was valued at the level of "Health centre (no beds)" using WHO-CHOICE outpatient visit unit costs. An ANC visit at a hospital was valued at the level of "Primary hospital" using WHO-CHOICE outpatient visit unit costs.
[b] Assumptions are based on those used in the WHO OneHealth costing tool.

The cost of an ANC visit was taken from the country-specific WHO-CHOICE estimates of the cost of an outpatient visit, 20% of which was attributed as a cost of the intervention. In many countries, community health workers are unsalaried, however, the economic costs of community health worker time was valued at country-specific minimum wage using estimates from the International Labour Organization. [22]

For iron supplementation, the per person cost of a point of care diagnostic test for anaemia (eg HemoCue) was estimated to be 0.50 USD based on a costing analysis conducted in India. [23] No costs have been applied to service delivery for supplements accessed through private retailers as these costs are borne by private industry and not included in the health system perspective adopted (unless passed on to consumers in the commodity price).

Service delivery cost estimates use several data sources and assumptions, each associated with considerable uncertainty. Rather than attempt to represent uncertainty within each of these parameters without reliable data to do so, in the sensitivity analysis we have used base case ± 50% for upper and lower estimates of service delivery costs as a whole.

To ensure highest possible coverage, distribution of insecticide treated bed nets is likely to occur through a combination of diverse channels, including school-based programs, mass campaigns and continuous health facility delivery. The channels adopted will vary between country settings and will be associated with different cost structures. To derive a unit cost estimate that best encompasses this variety in delivery strategies we drew from a systematic review and meta-analysis of the costs of insecticide-treated nets, taking the overall median economic cost per net distributed (Table A8). [24] This cost was taken to encompass both service delivery and program-level cost components, which is consistent with the methodology of the majority of the studies included in the meta-analysis. In the sensitivity analysis, this was varied from base case values by ± 1.96 times the standard error reported from this meta-analysis.

1.5.2. Interventions targeting non-pregnant women

In line with the approach used by Shekar and colleagues[25], we have assumed that supplements are delivered to this population through the combination of channels outlined in Table A7. We assume iron tablets are provided to non-pregnant women through 2 healthcare visits, in contrast to pregnant women who are assumed to access tablets over 4 ANC visits. Resource quantities and costs for provision of interventions to non-pregnant women were otherwise estimated as outlined for pregnant women.

Table A7: Inputs for estimating service delivery costs in non-pregnant women.

	World Bank Country-income classification			
	Low-income	*Lower-middle-income*	*Upper-middle-income*	*High-income*
Health facility visits[a]				
% of women below the poverty line accessing intervention through this channel[b]	30%	30%	30%	30%
% of women above the poverty line accessing intervention through this channel[b]	20%	20%	20%	30%
Number of visits for delivery of iron supplementation[c]	2	2	2	2
Number of visits for delivery of deworming tablets[c]	1	1	1	1
Proportion of visit dedicated to delivery of either iron or deworming tablets[c]	100%	100%	100%	100%
Community health worker visits				
% of women below the poverty line accessing intervention through this channel[c]	70%	70%	70%	0
% of women above the poverty line accessing intervention through this channel[c]	50%	50%	50%	0
Number of visits for delivery of iron supplementation[c]	2	2	2	n.a.
Number of visits for delivery of deworming tablets[c]	1	1	1	n.a.
Proportion of visit dedicated to delivery of either iron or deworming tablets[c]	5 mins	5 mins	5 mins	n.a.
Private pharmacies				
% of women below the poverty line accessing intervention through this channel[b]	0	0	0	70%
% of women above the poverty line accessing intervention through this channel[b]	30%	30%	30%	70%

[a] Health facility visits were valued at the level of "Health centre (no beds)" using WHO-CHOICE outpatient visit unit costs.
[b] Assumptions are broadly based on those used in the World Bank Investment Framework for Nutrition. [26]
[c] Assumptions are based on those used in the WHO OneHealth costing tool.

1.6. Program costs

The service delivery costs for insecticide treated bed nets (see above) were assumed to encompass all costs and no further program costs were added to the unit costs of this intervention.

For all other interventions the program level cost (per woman treated) were drawn from a study by Baltussen and colleagues, who modelled the start-up and ongoing costs of implementing a 10-year program of iron supplementation in four subregions (see Table A8).[27] This study adopted WHO-CHOICE methodology in the estimation of resource use and the associated costs.[28] Unit costs assume 95% coverage in the population. Program unit costs for countries in regions not included in the publication were drawn from the population-weighted average program unit costs in countries with equivalent income classification.

A lower estimate of 50% of these unit costs was used in a sensitivity analysis. For the upper estimate we took the total program costs but applied this to 50% coverage of the population (deriving a higher per person unit cost).

Table A8: Inputs for valuing program costs.

	Base case value (lower estimate, upper estimate)[a]	Source
Iron supplementation, intermittent antimalarials and deworming ($I 2000)		
AFR	0·49 (0·25, 0·92)	Baltussen et al.[29]; See note[b]
AMR	0·61 (0·31, 1·15)	Baltussen et al.[29]; See note[b]
EUR	4·12 (2·06, 7·83)	Baltussen et al.[29]; See note[b]
SEAR	0·30 (0·15, 0·57)	Baltussen et al.[29]; See note[b]
Insecticide treated bed nets (USD 2016)[c]		
Low-income	3·97 (3·49, 4·45)	Wisniewski, J., et al.[24]; See note[d]
Middle and high-income	6·88 (5·84, 7·92)	Wisniewski, J., et al.[24]; See note[d]

$I = International dollars; AFR = African region; AMR = Region of the Americas; EMR = Eastern Mediterranean region; EUR = European region; SEAR = South-East Asian region; USD = United States dollars; WPR = Western Pacific Region

[a] Costs reported here were first converted to local currency units (LCU) using global exchange rates relevant to the year of data (or using PPP conversion factor if source data was international dollars), then adjusted for inflation in the LCU using GDP implicit price deflators (giving 2023 LCU), before finally converting to USD using 2023 exchange rates (or PPP conversion factor if source data was international dollars).

[b] Base: Per person program costs at 95% population coverage reported in Baltussen et al, (2004)[29]. Low: 50% of base case. High: Total costs reported in Baltussen et al applied to 50% population coverage.

[c] This parameter was used to encompass both service delivery and program costs.

[d] Base: Median economic cost per net reported in Wisniewski, J., et al. (2020)[24]. Low and High: Median economic cost reported in Wisniewski, J., et al. (2020) ± 1·96 × standard error.

1.7. Staple food fortification

The unit cost (per person reached) of maize and wheat flour fortification was taken from Fiedler and colleagues who used an ingredients-based approach to estimate the total public and private sector costs of a national program of maize

flour fortification in 10 low- and lower-middle-income and 3 upper-middle income countries. [30] Total costs were to unit costs by dividing by 90% of the 2023 population in each country where costs were quantified (Table A9).

For maize fortification in low- and lower-middle-income countries, in the base case we use a population weighted average of this unit cost across the LMICs. For the sensitivity analysis we used the lowest unit cost estimate (0·09 USD per person based on cost estimates in Uganda) and the highest estimate (0·95 USD based on costs estimates in Bolivia). For high-income countries, where fewer cost estimates were available, we used the cost estimate from Guatemala (1.94 USD per person) for the base case, the estimate from South Africa (1.57 USD per person) as the low estimate and the estimate from Mexico (2.81 USD per person) as the high estimate.

For wheat flour fortification, in the base case we use a population weighted average of this unit cost, disaggregating low- and lower-middle-income countries from upper-middle-income countries. For the sensitivity analysis we used the lowest and highest unit cost estimate from these country groupings.

For rice fortification, we used unit cost estimates reported in a study by Alavi and colleagues, [31] which were adjusted for inflation (from 2008 USD) using the United States GDP inflater. In all countries, we used a unit cost of 0·74 USD per person based on the estimated costs to consumers of cold extrusion rice fortification in Costa Rica. For the sensitivity analysis, the lower estimate was based on the cost of coating methods of rice fortification in the United States (0·11 USD per person) and the upper estimate was based on hot extrusion fortification costs in China (2·26 USD per person).

We assume that the decision to adopt fortification of either (or both) staple food in each country would be based on a variety of factors, including dietary consumption and unit costs were calculated for settings where daily intake/availability of the staple food was greater than 75 g/c/d. In settings where intake was greater than 75 g/c/d for multiple staple foods, we calculated an average unit cost, weighted by the relative intake/consumption of each staple.

Table A9: Inputs for valuing fortification of maize and wheat flour. All costs drawn from Fiedler et al 2009. [30]

UN Member State	World Bank Income classification	Cost per person per year (USD 2008)	
		Wheat flour	*Maize*
**Afghanistan**	Low-income	0·0054	
**Burkina Faso**	Low-income	0·0093	
**DR Congo**	Low-income	0·0020	
**Ethiopia**	Low-income	0·0069	
**Madagascar**	Low-income	0·0060	
**Malawi**	Low-income	0·0102	0·2117
**Mali**	Low-income	0·0094	
**Mozambique**	Low-income	0·0080	

**Niger**	Low-income	0·0087	
**Sudan**	Low-income	0·0094	
**Uganda**	Low-income		0·0862
**Yemen**	Low-income	0·0084	
**Angola**	Lower-middle-income	0·0070	0·2125
**Bangladesh**	Lower-middle-income	0·0072	
**Bolivia**	Lower-middle-income		0·9546
**Cambodia**	Lower-middle-income	0.0088	
**Cameroon**	Lower-middle-income	0.0063	
**Myanmar**	Lower-middle-income	0.0025	
**Nepal**	Lower-middle-income	0.0083	
**Cote d'Ivoire**	Lower-middle-income	0.0066	0·1929
**Ghana**	Lower-middle-income	0.0068	0·2466
**Guinea**	Lower-middle-income	0.0119	
**India—low**	Lower-middle-income	0.0075	
**India -- high**	Lower-middle-income	0.0020	
**India**	Lower-middle-income	0.0094	
**Kenya**	Lower-middle-income	0.0041	0·3930
**Nigeria**	Lower-middle-income	0.0014	
**Pakistan—low**	Lower-middle-income	0.0208	
**Pakistan—high**	Lower-middle-income	0.0057	
**Pakistan**	Lower-middle-income	0.0265	
**Philippines**	Lower-middle-income	0.0030	
**Tanzania**	Lower-middle-income	0.0034	0·1721
**Uzbekistan**	Lower-middle-income	0.0116	
**Vietnam**	Lower-middle-income	0.0025	
**Zambia**	Lower-middle-income	0.0125	0·5307
**Zimbabwe**	Lower-middle-income	0.0152	0·6358
**Guatemala**	Upper-middle-income	0.0148	1·9369
**Mexico**	Upper-middle-income	0.0108	2·8050
**Indonesia**	Upper-middle-income	0.0008	
**Peru**	Upper-middle-income	0.0064	
**Turkey**	Upper-middle-income	0.0614	
**China**	Upper-middle-income	0.0408	
**South Africa**	Upper-middle-income	0.0041	1·5700
**Brazil**	Upper-middle-income	0.0056	

USD = United Stated dollars

See Table A10 for two case examples of how all inputs described above were used to estimate intervention unit costs.

A summary of the parameter inputs that were varied in these sensitivity analyses is shown in Table A11.

Table A10: Case study examples of how inputs are used to estimate unit costs.

	**Example case 1:** Iron supplementation for non-pregnant women in a high-income country	**Example case 2:** Intermittent preventative treatment of malaria in pregnancy in a lower-middle-income country
Commodity cost (A)	25·31 USD[a]	0·4489 USD
% of women anaemic (B)	22·84%	n.a
Cost of treatment dosing (C)	18·28 USD[b]	n.a
Cost of preventative dosing (D)	21·14 USD[c]	0·4489 USD[d]
Private sector mark-up (E)	n.a (assumed to be included in private sector commodity cost)	n.a (assumed to be delivered through public sector channels)
Supply cost (F)	n.a	0·13 USD
Mark-up (G)	n.a (assumed to be included in private sector commodity cost)	30%
Service delivery cost (H)[e]	10·67 USD	1·41 USD
% of women accessing through facility visit (I)	30%	36·9%[f]
% of women accessing through CHW (J)	0%	63·1%
% of women accessing through private pharmacy (K)	70%	0%
Cost per woman accessing through facility visit (L)	3390 USD[g]	1·04 USD[h]
Cost per woman accessing through CHW (M)	n.a	1·63 USD[i]
Cost per woman accessing through private pharmacy (N)	0 USD	0 USD
Cost of a haemoglobin test (O)	0·50 USD	n.a
Program cost (P)	3·63 USD	0·19 USD
Total unit cost (A + F + H + P)	39·61 USD	2·18 USD

CHW = community health worker; n.a. = not applicable; USD = United States dollars (2023)

[a] Weighted average costs accounting for proportion of women receiving treatment dosing schedule and proportion of women accessing supplements through private pharmacies according to the following formula: (1 - K) * (B * [C + D] + [1- B] * D) + K * (1 + E) * (B * [C + D] + [1- B] * D).

[b] 0·1998 USD per dose multiplied by 30·5 daily doses per month multiplied by three months for treatment of anaemia.

[c] 0·28 USD per dose multiplied by three doses per week multiplied by 4·2 weeks per month multiplied by six months for intermittent dosing (two cycles of 3 months on supplementation + 3 months off supplementation).

[d] 0·0397 per tablet multiplied by three tablets per dose multiplied by three doses for a full course.

[e] Weighted average costs accounting for proportion of women receiving intervention through each delivery channel according to the following formula: (I * L) + (J * M) + (K * N). Haemoglobin testing costs (O) are added to iron supplementation interventions for all women.

[f] Based on coverage of at least four ANC visits, 60% of which are assumed to take place at health centres and 40% at hospitals.

[g] Comprising two health centre visits at 16·95 USD each visit, assuming entire visit dedicated to delivery of the intervention.

[h] Weighted average of costs accounting for the proportion of ANC visits at health centres (1·60 USD per visit) and hospitals (2·11 USD per visit) multiplied by 20% of the visit dedicated to delivery of the intervention, multiplied by three visits for administration of full dosing course.

[i] Minimum hourly wage (6.50 USD/hr) for three visits with a community health worker at five minutes each visit.

Table A11: Parameter inputs varied in sensitivity analyses.

Parameter and intervention	Source for upper estimate	Source for lower estimate
**Commodity costs**		
Iron supplementation tablets	HIC: see note[a]. LMIC: Highest price from International Drug Price Indicator Guide.	HIC: see note[a]. LMIC: Lowest price from International Drug Price Indicator Guide.
Multiple micronutrient supplementation tablets	HIC: Private market (Walgreens). LMIC: Highest price from UNICEF supply catalogue.	HIC: Private market (Boots). LMIC: Lowest price from UNICEF supply catalogue.
Sulfadoxine 500 mg, Pyrimethamine 25 mg	Highest price from International Drug Price Indicator Guide.	Lowest price from International Drug Price Indicator Guide.
Insecticide treated bed nets (ITN)	Global Fund pricing catalogue.	UNICEF pricing catalogue.
Deworming tablets	HIC: see note[a]	HIC: see note[a]
	LMIC: Highest price from International Drug Price Indicator Guide	LMIC: Lowest price from International Drug Price Indicator Guide
Private sector mark-up on commodity price	Reported markup on pharmaceuticals in settings with highly regulated pharmaceutical pricing.[37]	Highest reported mark up in a setting with limited pharmaceutical pricing regulations.[38]
Supply costs		
All interventions	Stenberg, K., et al.[39]	Highest WHO-CHOICE mark-up
Service delivery costs		
All interventions except ITN	Base case + 50%	Base case - 50%
Program costs		
All interventions except ITN	Estimates from Baltussen et al, scaled for 50% coverage instead of 95%. [13]	Base case - 50%
Service delivery + program costs		
ITN	Base case + 1·96 × standard error reported from meta-analysis.[12]	Base case – 1·96 × standard error reported from meta-analysis.[12]
Total unit cost		
Wheat and maize flour fortification	High- and upper-middle-income countries: lowest country-specific estimate among data available for countries in these income groups (South Africa). Low- and lower-middle-income countries: lowest country-specific estimate among data available for countries in these income groups (Uganda)	High- and upper-middle-income countries: highest country-specific estimate among data available for countries in these income groups (Mexico). Low- and lower-middle-income countries: highest country-specific estimate among data available for countries in these income groups (Bolivia)
Rice fortification	Lowest estimate available for non-dusting methods of rice fortification (United States).	High estimate available for non-dusting methods of rice fortification (China).

HIC = High-income countries; LMIC = Low- and lower-middle-income-countries
[a] Three data sources were drawn on to estimate dose prices: UK pharmaceutical electronic market information tool (representing average price paid by public sector hospitals in the UK), prices listed by US-based private retailers (e.g. Walgreens), and estimated dispensed price estimated by the Australian Pharmaceutical Benefits Scheme. The median of the three sources was used as the base case, while the lowest and highest were used for the lower and upper estimates respectively.

1.8. Adjusting for inflation and currency conversion

All costs were reported in 2023 USD, with adjustments for inflation and currency conversions made as outlined by Turner and colleagues[32] and recommended by the Global Health Cost Consortium[33], which applies differential methodology for tradable and non-tradable goods. Tradable goods are those which can be imported or exported (such

as supplements and pharmaceuticals) and are therefore priced in line with global markets. Costs of tradable goods, were first converted from LCU to USD using exchange rates relevant for the year of data and then adjusted for inflation using the US inflation rate (which typically reflects changes in global market prices). Non-tradable goods on the other hand are those which must be consumed locally, such as staff salaries and overhead costs, and therefore are affected by local inflation rates. Costs of non-tradable goods were first converted to local currency units (LCU) using official exchange rates[34] relevant to the year of data or purchasing power parity (PPP) conversion factor if source data was international dollars. Costs in LCU were then adjusted for inflation to 2023 using GDP implicit price deflators, before finally converting to USD using 2023 exchange rates (or PPP conversion factor if source data was international dollars).

2. Detailed results

2.1. Country-specific unit costs

Table A12: Country-specific unit cost estimates for nutrition-specific interventions. Upper and lower estimates represent results of the 'extreme scenario analysis', where the maximum uncertainty associated with the unit cost of each intervention was explored by calculating unit costs using the lower or upper estimates for all cost components at the same time.

	**Base case (lower estimate, upper estimate), 2023 USD**			
UN Member State	*Oral iron & folic acid supplementation for pregnant women*	*Oral iron & folic acid supplementation for non-pregnant women*	*Staple food fortification*	*Multiple micronutrients*
Afghanistan	2·25 (0·96, 4·11)	2·15 (1·1, 5·25)	0·01 (·<0·01, 0·04)	8·82 (5·22, 10·47)
Albania	10·7 (5·27, 17·07)	7·9 (3·98, 14·52)	0·04 (·<0·01, 0·04)	16·66 (9·51, 23·36)
Algeria	4·75 (2·17, 7·81)	3·72 (1·83, 8·13)	0·01 (·<0·01, 0·04)	11·83 (6·39, 14)
Andorra	88·26 (33·25, 138·56)	n.a.	n.a.	141·48 (88·34, 209·6)
Angola	4·89 (2·29, 8·23)	4·46 (2·68, 11·82)	0·3 (0·13, 1·33)	11·95 (6·45, 14·18)
Antigua and Barbuda	63·48 (20·73, 100·99)	37·19 (10·52, 53·66)	0·04 (·<0·01, 0·04)	114·3 (74·75, 168·83)
Argentina	17·59 (8·72, 27·47)	n.a.	n.a.	23·95 (12·97, 33·74)
Armenia	10·47 (5·14, 16·65)	7·16 (3·61, 13·31)	0·04 (·<0·01, 0·04)	16·44 (9·4, 23·03)
Australia	109·33 (43·79, 170·08)	n.a.	0·04 (·<0·01, 0·04)	162·56 (98·88, 241·22)
Austria	57·37 (17·8, 92·24)	n.a.	0·04 (·<0·01, 0·04)	110·59 (72·89, 163·27)
Azerbaijan	12·81 (6·34, 20·32)	9·4 (4·74, 16·87)	0·04 (·<0·01, 0·04)	18·76 (10·56, 26·51)
Bahamas	85·42 (31·72, 133·62)	53·08 (18·45, 77·75)	n.a.	136·29 (85·74, 201·81)
Bahrain	62·54 (20·36, 98·49)	36·06 (12·48, 66·84)	n.a.	113·57 (74·38, 167·74)
Bangladesh	3·67 (1·74, 6·56)	3·13 (2·07, 10·64)	0·76 (0·11, 2·33)	10·05 (5·94, 12·63)
Barbados	65·95 (21·93, 105·05)	39·76 (11·83, 57·18)	0·04 (·<0·01, 0·04)	116·7 (75·95, 172·43)
Belarus	83·37 (41·58, 125·97)	35·36 (17·71, 55·59)	0·04 (·<0·01, 0·04)	89·34 (45·85, 132·38)
Belgium	57·38 (17·8, 92·32)	n.a.	0·04 (·<0·01, 0·04)	110·59 (72·89, 163·27)
Belize	9·85 (4·84, 15·84)	5·8 (3·42, 14·78)	0·04 (·<0·01, 0·04)	16·1 (9·05, 21·96)
Benin	2·77 (1·26, 5·23)	2·92 (1·86, 9·83)	0·61 (0·12, 1·97)	9·81 (5·38, 10·98)
Bhutan	4·18 (2·03, 7·48)	3·25 (2·1, 10·85)	0·76 (0·11, 2·33)	10·54 (6·18, 13·37)
Bolivia	5·32 (2·53, 8·8)	3·75 (1·88, 8·12)	0·33 (0·05, 1·02)	11·58 (6·79, 15·19)
Bosnia and Herzegovina	11 (5·41, 17·45)	7·95 (4, 14·52)	0·04 (·<0·01, 0·04)	16·96 (9·66, 23·81)

Botswana	9·5 (4·55, 14·92)	6·31 (3·13, 11·83)	1·82 (1·47, 2·63)	16·58 (8·77, 21·13)
Brazil	3·84 (1·82, 6·74)	3·13 (1·58, 7·34)	0·01 (·<0·01, 0·04)	10·09 (6·04, 12·95)
Brunei Darussalam	68·27 (23·3, 107·95)	n.a.	0·76 (0·11, 2·33)	121·54 (78·36, 179·68)
Bulgaria	16·22 (8·02, 25·3)	10·67 (5·37, 18·62)	0·04 (·<0·01, 0·04)	22·18 (12·27, 31·64)
Burkina Faso	2·75 (1·2, 4·96)	2·71 (1·81, 9·36)	0·35 (0·13, 1·33)	9·81 (5·38, 10·97)
Burundi	2·31 (0·96, 4·18)	2·26 (1·14, 5·28)	n.a.	9·38 (5·17, 10·33)
Cabo Verde	5·24 (2·43, 8·62)	3·97 (2·44, 11·77)	0·56 (0·12, 1·85)	12·31 (6·63, 14·72)
Cambodia	3·12 (1·5, 5·82)	2·73 (1·9, 9·97)	0·76 (0·11, 2·33)	9·18 (5·7, 11·92)
Cameroon	3·21 (1·41, 5·53)	2·91 (1·44, 6·69)	0·53 (0·12, 1·77)	10·29 (5·62, 11·69)
Canada	99·85 (39·08, 155·5)	n.a.	0·04 (·<0·01, 0·04)	153·1 (94·15, 227·03)
Central African Republic	2·63 (1·12, 4·67)	2·72 (1·38, 5·92)	n.a.	9·71 (5·33, 10·82)
Chad	2·49 (1·1, 4·7)	2·86 (1·86, 9·38)	n.a.	9·53 (5·25, 10·56)
Chile	56·17 (17·2, 90·41)	n.a.	0·04 (·<0·01, 0·04)	109·39 (72·29, 161·47)
China	8·89 (4·39, 14·47)	n.a.	0·5 (0·07, 1·48)	15·07 (8·65, 20·76)
Colombia	12·75 (6·29, 20·16)	n.a.	1·65 (1·07, 3·06)	19·12 (10·56, 26·5)
Comoros	3·04 (1·32, 5·28)	2·79 (1·38, 6·56)	0·76 (0·11, 2·33)	10·11 (5·54, 11·43)
Congo	5·29 (2·48, 8·78)	4·38 (2·65, 11·63)	0·01 (·<0·01, 0·04)	12·35 (6·66, 14·79)
DR Congo	2·64 (1·1, 4·57)	2·62 (1·33, 5·55)	n.a.	9·73 (5·34, 10·85)
Costa Rica	16·51 (8·18, 25·86)	n.a.	0·44 (0·06, 1·29)	22·87 (12·43, 32·12)
Croatia	59·13 (18·71, 92·64)	34·29 (11·45, 64·33)	0·04 (·<0·01, 0·04)	110·3 (72·75, 162·83)
Cuba	8·84 (4·35, 14·38)	n.a.	0·48 (0·07, 1·42)	15·2 (8·6, 20·62)
Cyprus	116·58 (47·25, 181·01)	74·75 (29·33, 109·64)	0·04 (·<0·01, 0·04)	167·33 (101·26, 248·38)
Czechia	71·18 (24·71, 112·89)	n.a.	0·04 (·<0·01, 0·04)	124·4 (79·8, 183·98)
Cote d'Ivoire	3·03 (1·35, 5·36)	n.a.	0·76 (0·11, 2·33)	10·23 (5·6, 11·61)
Denmark	109·89 (44·07, 169·09)	69·11 (28·92, 116·5)	0·04 (·<0·01, 0·04)	161·01 (98·1, 238·89)
Djibouti	2·79 (1·27, 5·07)	n.a.	0·32 (0·05, 0·97)	9·48 (5·55, 11·46)
Dominica	14·71 (7·26, 23·08)	8·44 (4·24, 15·38)	0·04 (·<0·01, 0·04)	20·95 (11·47, 29·24)
Dominican Republic	9·8 (4·8, 15·68)	5·5 (2·77, 10·94)	0·76 (0·11, 2·33)	16·05 (9·03, 21·9)
Ecuador	6·55 (3·17, 10·8)	5·2 (2·62, 10·46)	1·18 (0·77, 2·14)	12·8 (7·4, 17·02)
Egypt	4·91 (2·33, 8·29)	n.a.	0·21 (0·05, 0·72)	11·6 (6·6, 14·63)
El Salvador	2·57 (1·16, 4·74)	5·63 (2·83, 10·99)	2·69 (2·18, 3·9)	8·83 (5·41, 11·07)
Equatorial Guinea	13·92 (6·77, 21·59)	n.a.	n.a.	21·14 (11·05, 27·97)
Eritrea	2·29 (0·97, 4·27)	2·39 (1·66, 8·45)	0·01 (·<0·01, 0·04)	9·35 (5·16, 10·29)
Estonia	42·84 (10·49, 69·08)	24·29 (6·62, 49·15)	n.a.	93·84 (64·52, 138·14)
Eswatini	4·31 (1·99, 7·28)	n.a.	0·35 (0·13, 1·33)	11·52 (6·24, 13·53)
Ethiopia	2·62 (1·09, 4·53)	2·55 (1·25, 5·99)	0·22 (0·08, 0·83)	9·7 (5·33, 10·82)
Fiji	6·91 (3·35, 11·27)	4·76 (2·4, 9·7)	0·37 (0·05, 1·08)	12·99 (7·61, 17·64)
Finland	59·68 (18·87, 94·81)	38·23 (11·01, 55·67)	0·04 (·<0·01, 0·04)	110·59 (72·89, 163·27)
France	58·64 (18·43, 94·2)	n.a.	0·04 (·<0·01, 0·04)	111·86 (73·53, 165·17)
Gabon	10·39 (5·01, 16·28)	n.a.	0·37 (0·05, 1·09)	17·62 (9·29, 22·69)
Gambia	2·82 (1·26, 5·19)	2·67 (1·76, 9·42)	0·76 (0·11, 2·33)	9·87 (5·42, 11·07)
Georgia	8·15 (4·07, 13·6)	6·61 (3·82, 15·88)	0·04 (·<0·01, 0·04)	14·08 (8·22, 19·49)
Germany	61·28 (19·54, 98·63)	37·28 (10·63, 52·89)	0·04 (·<0·01, 0·04)	111·91 (73·55, 165·24)
Ghana	3·39 (1·52, 5·84)	n.a.	0·76 (0·11, 2·33)	10·61 (5·78, 12·18)
Greece	39·87 (9·18, 62·68)	24·15 (6·16, 49·33)	0·04 (·<0·01, 0·04)	91·25 (63·22, 134·26)
Grenada	12·66 (6·24, 20)	n.a.	n.a.	19·04 (10·52, 26·37)
Guatemala	6·48 (3·14, 10·69)	4·42 (2·24, 9·21)	2·69 (2·18, 3·9)	12·74 (7·37, 16·92)

Guinea	2·81 (1·2, 4·87)	2·64 (1·29, 6·33)	0·76 (0·11, 2·33)	9·89 (5·42, 11·09)
Guinea-Bissau	2·96 (1·31, 5·27)	2·82 (1·87, 9·52)	0·76 (0·11, 2·33)	10·03 (5·49, 11·3)
Guyana	44·89 (11·67, 70·39)	27·46 (7·85, 54·27)	0·51 (0·07, 1·51)	96·23 (65·71, 141·73)
Haiti	3·47 (1·64, 6·23)	3·08 (1·59, 7·04)	0·76 (0·11, 2·33)	9·72 (5·86, 12·39)
Honduras	5·72 (2·82, 9·86)	4·36 (2·67, 12·25)	0·35 (0·13, 1·33)	11·94 (6·97, 15·73)
Hungary	60·08 (19·23, 95·43)	n.a.	0·04 (·<0·01, 0·04)	113·37 (74·28, 167·43)
Iceland	115·25 (46·48, 180·13)	71·83 (27·94, 104·2)	n.a.	165·78 (100·48, 246·04)
India	2·61 (1·19, 4·84)	n.a.	0·42 (0·06, 1·27)	9·13 (5·48, 11·26)
Indonesia	3·8 (1·84, 6·9)	3·07 (2·01, 10·54)	0·76 (0·11, 2·33)	10·17 (5·99, 12·8)
Iran	41·87 (20·78, 63·59)	19·4 (9·7, 31·65)	0·04 (·<0·01, 0·04)	48·44 (25·02, 69·89)
Iraq	4·66 (2·18, 7·78)	3·85 (1·92, 8·32)	0·21 (0·03, 0·56)	11·23 (6·42, 14·08)
Ireland	59·83 (18·88, 95·81)	37·71 (10·81, 54·16)	0·04 (·<0·01, 0·04)	110·59 (72·89, 163·27)
Israel	88·21 (33·22, 138·53)	n.a.	0·04 (·<0·01, 0·04)	141·43 (88·31, 209·53)
Italy	54·76 (16·51, 88·13)	n.a.	0·04 (·<0·01, 0·04)	107·99 (71·59, 159·37)
Jamaica	9·53 (4·67, 15·28)	n.a.	0·04 (·<0·01, 0·04)	15·9 (8·95, 21·67)
Japan	75·91 (26·96, 119·38)	47·05 (15·44, 68·69)	0·5 (0·07, 1·49)	126·78 (80·99, 187·54)
Jordan	5·14 (2·46, 8·68)	n.a.	0·01 (·<0·01, 0·04)	11·83 (6·72, 14·97)
Kazakhstan	12·7 (6·3, 20·23)	8·18 (4·63, 18·45)	0·04 (·<0·01, 0·04)	18·65 (10·51, 26·34)
Kenya	3·04 (1·33, 5·29)	2·83 (1·41, 6·42)	0·55 (0·13, 1·33)	10·12 (5·54, 11·44)
Kiribati	2·21 (1, 4·24)	3·21 (1·63, 7·39)	0·54 (0·08, 1·67)	8·29 (5·26, 10·6)
Kuwait	60·85 (19·55, 95·47)	36·43 (12·57, 67·49)	0·35 (0·05, 1·01)	111·97 (73·58, 165·34)
Kyrgyzstan	5·78 (2·81, 9·67)	4·97 (2·52, 10·1)	0·01 (·<0·01, 0·04)	11·74 (7·05, 15·98)
Laos	2·67 (1·26, 5·09)	2·46 (1·27, 6·38)	0·76 (0·11, 2·33)	8·74 (5·48, 11·26)
Latvia	62·62 (20·45, 97·97)	40·08 (14·36, 73)	0·04 (·<0·01, 0·04)	113·78 (74·49, 168·05)
Lebanon	87·96 (43·81, 132·63)	56·52 (28·25, 87·23)	0·01 (·<0·01, 0·04)	94·54 (48·07, 139·04)
Lesotho	2·97 (1·25, 4·99)	2·5 (1·23, 5·8)	0·35 (0·13, 1·33)	10·06 (5·51, 11·35)
Liberia	2·06 (0·81, 3·69)	1·81 (0·89, 4·88)	0·76 (0·11, 2·33)	9·14 (5·05, 9·98)
Libya	9·48 (4·69, 15·55)	6·45 (3·65, 15·48)	0·04 (·<0·01, 0·04)	16 (8·8, 21·23)
Lithuania	65·11 (21·54, 103·49)	40·05 (11·96, 57·9)	0·04 (·<0·01, 0·04)	115·92 (75·56, 171·26)
Luxembourg	61·13 (19·42, 98·96)	36·92 (10·48, 51·83)	0·04 (·<0·01, 0·04)	111·66 (73·43, 164·87)
Madagascar	2·17 (0·9, 3·95)	n.a.	0·76 (0·11, 2·33)	9·4 (5·18, 10·35)
Malawi	2·93 (1·26, 5·09)	2·84 (1·44, 6·04)	0·31 (0·13, 1·33)	10·01 (5·48, 11·27)
Malaysia	10·54 (5·26, 17·18)	6·3 (3·64, 15·49)	0·55 (0·08, 1·67)	16·58 (9·4, 23·03)
Maldives	9·99 (4·95, 16·3)	6·22 (3·56, 15·26)	0·47 (0·07, 1·4)	16·35 (9·08, 22·07)
Mali	2·56 (1·14, 4·82)	2·75 (1·8, 9·44)	0·56 (0·12, 1·84)	9·6 (5·28, 10·66)
Malta	135·76 (57·14, 206·33)	88·43 (38·26, 145·79)	0·04 (·<0·01, 0·04)	187·18 (111·19, 278·15)
Marshall Islands	7·95 (3·91, 12·99)	n.a.	0·32 (0·04, 0·91)	14·14 (8·18, 19·37)
Mauritania	8·24 (3·92, 13·05)	9·05 (4·5, 16·07)	0·28 (0·04, 0·86)	15·32 (8·14, 19·24)
Mauritius	10·67 (5·17, 16·88)	6·38 (3·62, 15·42)	0·31 (0·04, 0·89)	17·73 (9·34, 22·86)
Mexico	17·8 (8·8, 27·68)	8·98 (4·51, 16·16)	3·9 (2·18, 3·9)	24·04 (13·02, 33·88)
Micronesia	6·34 (3·1, 10·59)	n.a.	0·76 (0·11, 2·33)	12·53 (7·38, 16·95)
Republic of Moldova	8·97 (4·44, 14·62)	7·17 (4·13, 16·94)	0·92 (0·72, 1·31)	14·92 (8·64, 20·74)
Monaco	57·38 (17·8, 92·4)	n.a.	n.a.	110·59 (72·89, 163·27)
Mongolia	4·25 (2·03, 7·34)	3·07 (1·56, 7·23)	0·01 (·<0·01, 0·04)	10·32 (6·27, 13·64)
Montenegro	18·49 (9·15, 28·66)	11·48 (5·77, 19·77)	0·04 (·<0·01, 0·04)	24·45 (13·41, 35·05)
Morocco	4·19 (1·95, 7·15)	3·77 (1·88, 8·28)	0·08 (0·03, 0·3)	10·75 (6·18, 13·35)
Mozambique	2·48 (1·09, 4·68)	2·66 (1·8, 8·1)	0·35 (0·13, 1·33)	9·53 (5·25, 10·56)

Myanmar	2·9 (1·39, 5·56)	2·81 (1·87, 10·14)	0·76 (0·11, 2·33)	9·26 (5·54, 11·45)
Namibia	5·32 (2·44, 8·6)	4·18 (2·06, 8·57)	1·42 (1·14, 2·05)	12·4 (6·68, 14·86)
Nauru	46·99 (12·58, 75·16)	28·21 (8·56, 55·06)	n.a.	98·02 (66·61, 144·41)
Nepal	2·64 (1·24, 5·08)	2·5 (1·74, 9·58)	0·49 (0·09, 1·56)	9·01 (5·41, 11·06)
Netherlands	57·37 (17·8, 92·24)	n.a.	0·04 (·<0·01, 0·04)	110·59 (72·89, 163·27)
New Zealand	86·8 (32·53, 136·3)	n.a.	0·04 (·<0·01, 0·04)	140·03 (87·61, 207·43)
Nicaragua	4·28 (2·07, 7·5)	n.a.	0·52 (0·12, 1·76)	10·65 (6·32, 13·79)
Niger	2·33 (1, 4·37)	2·52 (1·72, 8·47)	n.a.	9·38 (5·17, 10·33)
Nigeria	3·26 (1·49, 5·87)	3·17 (2·01, 9·84)	0·55 (0·12, 1·81)	10·31 (5·63, 11·72)
North Korea	1·88 (0·84, 3·78)	1·91 (1·02, 5·03)	0·63 (0·12, 2·01)	7·96 (5·09, 10·09)
North Macedonia	11·36 (5·58, 17·96)	7·47 (3·76, 13·73)	0·04 (·<0·01, 0·04)	17·33 (9·85, 24·37)
Norway	132·4 (55·31, 204·88)	n.a.	0·04 (·<0·01, 0·04)	185·62 (110·41, 275·8)
Oman	59·03 (18·5, 94·35)	37·56 (10·71, 54·17)	0·42 (0·06, 1·23)	109·84 (72·51, 162·13)
Pakistan	2·49 (1·16, 4·87)	2·59 (1·76, 9·71)	0·04 (·<0·01, 0·04)	9·03 (5·32, 10·77)
Palau	22·74 (11·3, 35·2)	12·75 (6·41, 21·88)	0·76 (0·11, 2·33)	28·81 (15·51, 41·36)
Panama	54·95 (16·35, 89·38)	32·53 (8·27, 45·53)	0·76 (0·11, 2·33)	105·53 (70·36, 155·68)
Papua New Guinea	3·81 (1·88, 7·01)	3·99 (2·53, 11·16)	0·01 (·<0·01, 0·04)	9·86 (6·04, 12·94)
Paraguay	5·64 (2·7, 9·37)	3·74 (1·89, 8·22)	2·69 (2·18, 3·9)	11·89 (6·94, 15·65)
Peru	10·32 (5·04, 16·36)	5·61 (2·82, 10·97)	0·76 (0·11, 2·33)	16·58 (9·29, 22·68)
Philippines	3·94 (1·88, 6·9)	3·02 (1·54, 7·16)	0·63 (0·09, 1·92)	10·01 (6·12, 13·17)
Poland	60·72 (19·23, 98·07)	37·2 (10·61, 52·5)	0·04 (·<0·01, 0·04)	111·3 (73·24, 164·32)
Portugal	57·36 (17·81, 92·08)	n.a.	0·04 (·<0·01, 0·04)	110·59 (72·89, 163·27)
Puerto Rico	107·05 (42·42, 167·29)	64·54 (24·26, 93·77)	n.a.	157·68 (96·44, 233·9)
Qatar	100·1 (39, 156·25)	63·02 (23·46, 92·07)	n.a.	150·85 (93·02, 223·65)
Romania	57·17 (17·48, 92·53)	36·32 (10·15, 51·4)	0·04 (·<0·01, 0·04)	107·79 (71·49, 159·07)
Russian Federation	18·29 (9·05, 28·38)	11·79 (5·92, 20·26)	0·04 (·<0·01, 0·04)	24·25 (13·31, 34·75)
Rwanda	2·39 (0·96, 4·11)	2·27 (1·13, 5·23)	n.a.	9·49 (5·22, 10.5)
Samoa	5·65 (2·78, 9·64)	n.a.	0·37 (0·05, 1·14)	11·83 (7·03, 15·9)
San Marino	100·82 (39·43, 156·67)	63·4 (23·61, 93·29)	n.a.	151·7 (93·45, 224·93)
Saudi Arabia	58·34 (18·2, 92·77)	36·95 (10·37, 53·77)	0·28 (0·04, 0·78)	109·25 (72·22, 161·26)
Senegal	2·65 (1·13, 4·71)	2·75 (1·35, 6·61)	0·55 (0·08, 1·68)	9·72 (5·34, 10·85)
Serbia	13·78 (6·81, 21·67)	8·62 (4·35, 15·57)	0·04 (·<0·01, 0·04)	19·74 (11·05, 27·98)
Seychelles	56·86 (17·54, 91·48)	n.a.	0·42 (0·06, 1·25)	110·08 (72·63, 162·49)
Sierra Leone	2·41 (1·02, 4·39)	2·32 (1·63, 8·8)	0·76 (0·11, 2·33)	9·48 (5·22, 10·48)
Singapore	98·02 (37·99, 152·84)	60·52 (22·19, 88·61)	0·44 (0·06, 1·31)	148·83 (92·01, 220·63)
Slovakia	40·67 (9·68, 62·75)	25·7 (6·71, 51·89)	0·04 (·<0·01, 0·04)	92·26 (63·73, 135·78)
Slovenia	40·78 (9·26, 68·26)	23·45 (3·74, 31·79)	0·04 (·<0·01, 0·04)	91·34 (63·27, 134·39)
Solomon Islands	4·7 (2·27, 8·11)	3·9 (2, 8·3)	0·76 (0·11, 2·33)	10·77 (6·49, 14·3)
Somalia	2·67 (1·22, 4·99)	3·18 (2·11, 9·78)	0·76 (0·11, 2·33)	9·22 (5·41, 11·06)
South Africa	7·33 (3·5, 11·81)	n.a.	1·37 (1·37, 2·46)	14·54 (7·75, 18·07)
South Korea	71·74 (24·73, 114·79)	41·89 (12·96, 59·37)	0·5 (0·07, 1·51)	122·28 (78·74, 180·8)
South Sudan	2·32 (0·99, 4·28)	n.a.	n.a.	9·52 (5·24, 10·55)
Spain	59·51 (18·86, 93·7)	36·58 (12·69, 67·66)	0·04 (·<0·01, 0·04)	110·59 (72·89, 163·27)
Sri Lanka	5·57 (2·72, 9·55)	3·9 (2·42, 11·81)	0·6 (0·09, 1·84)	11·94 (6·88, 15·46)
St· Kitts and Nevis	58·12 (18·06, 92·77)	35·03 (9·43, 50·58)	n.a.	108·97 (72·08, 160·83)
St· Lucia	16·02 (7·92, 25·03)	n.a.	0·04 (·<0·01, 0·04)	22·39 (12·19, 31·41)
St· Vincent and the Grenadines	12·72 (6·28, 20·16)	6·8 (3·91, 16·27)	0·04 (·<0·01, 0·04)	18·96 (10·48, 26·25)

Sudan	4·36 (2·05, 7·43)	n.a.	0·01 (·<0·01, 0·04)	11·06 (6·33, 13·82)
Suriname	6·52 (3·16, 10·78)	4·91 (2·48, 10·08)	0·5 (0·07, 1·5)	12·77 (7·38, 16·97)
Sweden	97·86 (38·03, 151·26)	60·44 (24·63, 103·44)	0·04 (·<0·01, 0·04)	148·93 (92·06, 220·78)
Switzerland	142·17 (60·15, 218·15)	88·33 (38·66, 145·2)	0·04 (·<0·01, 0·04)	193·16 (114·18, 287·12)
Syria	2·94 (1·34, 5·29)	n.a.	0·01 (·<0·01, 0·04)	9·64 (5·62, 11·69)
Sao Tome and Principe	4·51 (2·08, 7·52)	n.a.	0·41 (0·06, 1·24)	11·72 (6·34, 13·85)
Tajikistan	3·66 (1·77, 6·59)	3·51 (1·8, 7·97)	0·01 (·<0·01, 0·04)	9·61 (5·99, 12·79)
Tanzania	2·79 (1·21, 4·95)	2·7 (1·83, 8·94)	0·41 (0·12, 1·65)	9·87 (5·41, 11·06)
Thailand	6·78 (3·24, 10·96)	4·01 (2·01, 8·52)	0·76 (0·11, 2·33)	13·18 (7·5, 17·33)
Timor-Leste	3·35 (1·55, 5·93)	2·83 (1·44, 6·61)	0·62 (0·12, 1·98)	9·74 (5·78, 12·17)
Togo	2·49 (1·12, 4·81)	2·68 (1·75, 9·16)	0·35 (0·13, 1·33)	9·52 (5·24, 10·55)
Tonga	8·03 (3·94, 13·11)	5·67 (2·87, 11·26)	0·04 (·<0·01, 0·04)	14·1 (8·16, 19·3)
Trinidad and Tobago	66·37 (22·21, 104·96)	39·03 (11·42, 56·77)	0·04 (·<0·01, 0·04)	117·26 (76·23, 173·27)
Tunisia	5·82 (2·75, 9·51)	3·92 (1·95, 8·4)	0·01 (·<0·01, 0·04)	12·4 (7, 15·82)
Turkey	12·3 (6·08, 19·5)	7.77 (3.92, 14.36)	0·09 (·<0·01, 0·04)	18·26 (10·31, 25·76)
Turkmenistan	1·5 (0·69, 3·34)	1·83 (0·96, 5·48)	0·04 (·<0·01, 0·04)	7·46 (4·91, 9·55)
Tuvalu	7·07 (3·47, 11·74)	5·85 (3·46, 14·84)	0·76 (0·11, 2·33)	13·13 (7·68, 17·85)
Uganda	2·64 (1·11, 4·59)	2·47 (1·23, 5·74)	0·13 (0·13, 1·33)	9·72 (5·34, 10·84)
Ukraine	10·09 (5·01, 16·34)	n.a.	0·01 (·<0·01, 0·04)	16·15 (9·26, 22·6)
United Arab Emirates	74·41 (26·37, 115·4)	45·19 (16·86, 80·7)	0·04 (·<0·01, 0·04)	125·62 (80·41, 185·8)
United Kingdom	92·84 (35·54, 145·39)	n.a.	0·04 (·<0·01, 0·04)	146·06 (90·63, 216·47)
United States of America	117·63 (47·98, 180·27)	67·75 (28·15, 114·55)	0·04 (·<0·01, 0·04)	168·83 (102·01, 250·62)
Uruguay	59·31 (18·81, 94·7)	n.a.	0·04 (·<0·01, 0·04)	112·57 (73·88, 166·23)
Uzbekistan	4·08 (1·99, 7·26)	n.a.	0·02 (·<0·01, 0·04)	10·15 (6·26, 13·59)
Vanuatu	6·65 (3·3, 11·26)	6·2 (3·61, 15·15)	0·77 (0·11, 2·35)	12·7 (7·46, 17·21)
Venezuela	10·51 (5·2, 16·96)	6·63 (3·81, 15·82)	1·74 (1·16, 3·12)	16·74 (9·37, 22·93)
Vietnam	3·46 (1·68, 6·36)	n.a.	0·76 (0·11, 2·33)	9·63 (5·93, 12·6)
Yemen	2·21 (0·94, 4·07)	2·36 (1·19, 5·84)	0·01 (·<0·01, 0·04)	8·79 (5·2, 10·41)
Zambia	2·72 (1·22, 5·09)	2·72 (1·81, 8·39)	0·74 (0·13, 1·33)	9·76 (5·36, 10·9)
Zimbabwe	4·06 (1·84, 6·84)	3·7 (2·33, 10·56)	0·88 (0·13, 1·33)	11·13 (6·04, 12·95)

n.a. = not applicable. Unit costs have not been estimated for iron supplementation in non-pregnant women in settings where anaemia prevalence is < 20% because the World Health Organization does not make explicit recommendations for these settings. Unit costs for staple food fortification have only been estimated for countries where Daily Food Availability is greater than 75 grams per capita per day (g/c/d); USD = United Stated dollars

Table A13: Country-specific unit cost estimates for nutrition-sensitive interventions. Upper and lower estimates represent results of the 'extreme scenario analysis', where the maximum uncertainty associated with the unit cost of each intervention was explored by calculating unit costs using the lower or upper estimates for all cost components at the same time.

	Base case (lower estimate, upper estimate), 2023 USD			
**UN Member state**	*Intermittent preventive treatment with sulfadoxine-pyrimethamine*	*Deworming for pregnant women*	*Deworming for non-pregnant women*	*Insecticide treated bed nets*
Afghanistan	1·16 (0·63, 2·29)	n.a.	n.a.	5·77 (2·03, 3·82)
Angola	3·06 (1·56, 5·07)	n.a.	n.a.	7·49 (2·34, 4·67)
Bangladesh	2·18 (1·15, 3·85)	0·71 (0·37, 1·3)	0·83 (0·43, 1·82)	8·15 (2·53, 5·02)
Benin	1·44 (0·75, 2·64)	n.a.	n.a.	8·71 (2·65, 5·23)
Bolivia	3·51 (1·82, 5·85)	n.a.	n.a.	9·17 (2·79, 5·5)
Brazil	2·41 (1·27, 4·21)	n.a.	n.a.	8·45 (2·61, 5·17)
Burkina Faso	1·44 (0·75, 2·64)	n.a.	n.a.	5·74 (2, 3·77)
Burundi	1·12 (0·59, 2·16)	n.a.	n.a.	5·38 (1·9, 3·62)
Cambodia	1·82 (0·98, 3·35)	n.a.	n.a.	9 (2·76, 5·44)
Cameroon	1·81 (0·93, 3·19)	n.a.	n.a.	9 (2·72, 5·36)
Central African Republic	1·4 (0·73, 2·58)	n.a.	n.a.	5·97 (2·06, 3·87)
Chad	1·24 (0·65, 2·33)	0·45 (0·24, 0·9)	0·68 (0·35, 1·62)	6 (2·07, 3·89)
Colombia	9·15 (4·64, 14·32)	n.a.	n.a.	8·45 (2·61, 5·17)
Comoros	1·69 (0·87, 3·01)	n.a.	n.a.	8·99 (2·71, 5·36)
Congo	3·39 (1·72, 5·56)	n.a.	n.a.	11·72 (3·39, 6·61)
DR Congo	1·45 (0·75, 2·65)	n.a.	n.a.	7·11 (2·37, 4·37)
Cote d'Ivoire	1·76 (0·91, 3·12)	n.a.	n.a.	8·79 (2·67, 5·27)
Djibouti	1·63 (0·87, 3)	n.a.	n.a.	8·99 (2·74, 5·4)
Ecuador	4·43 (2·28, 7·23)	n.a.	n.a.	8·23 (2·56, 5·07)
Equatorial Guinea	9·96 (5·01, 15·41)	n.a.	n.a.	10·6 (3·12, 6·1)
Eritrea	1·09 (0·57, 2·11)	n.a.	n.a.	6·45 (2·19, 4·08)
Ethiopia	1·38 (0·72, 2·55)	n.a.	n.a.	6·67 (2·25, 4·18)
Gabon	7·32 (3·69, 11·46)	n.a.	n.a.	10·27 (3·03, 5·95)
Gambia	1·46 (0·76, 2·67)	n.a.	n.a.	5·64 (1·97, 3·73)
Ghana	2·1 (1·08, 3·62)	n.a.	n.a.	7·93 (2·45, 4·87)
Guinea	1·52 (0·79, 2·76)	n.a.	n.a.	12·76 (3·65, 7·08)
Guinea-Bissau	1·62 (0·84, 2·9)	0·6 (0·31, 1·12)	0·71 (0·37, 1·66)	5·86 (2·03, 3·83)
Guyana	4·46 (2·3, 7·26)	n.a.	n.a.	7·05 (2·16, 4·18)
Haiti	2·13 (1·13, 3·79)	n.a.	n.a.	11·87 (3·46, 6·74)
Honduras	3·81 (1·97, 6·3)	1·49 (0·76, 2·46)	1·6 (0·81, 2·98)	9·85 (2·96, 5·81)
India	1·48 (0·8, 2·79)	n.a.	n.a.	8·65 (2·66, 5·25)
Indonesia	2·26 (1·19, 3·96)	n.a.	n.a.	8·81 (2·7, 5·33)
Kenya	1·71 (0·88, 3·04)	n.a.	n.a.	8·38 (2·56, 5·08)
Laos	1·49 (0·82, 2·85)	n.a.	n.a.	6·2 (2·06, 4·16)
Liberia	0·87 (0·46, 1·79)	n.a.	n.a.	3·92 (1·51, 2·98)
Madagascar	1·11 (0·58, 2·15)	n.a.	n.a.	5·68 (1·99, 3·75)

Malawi	1·65 (0·85, 2·95)	n.a.	n.a.	10·17 (3·2, 5·71)
Mali	1·29 (0·67, 2·41)	0·46 (0·24, 0·91)	0·61 (0·32, 1·5)	5·78 (2·01, 3·79)
Mauritania	5·59 (2·83, 8·87)	n.a.	n.a.	10·15 (3, 5·89)
Mozambique	1·24 (0·65, 2·34)	0·45 (0·24, 0·9)	0·62 (0·33, 1·61)	6·79 (2·29, 4·23)
Myanmar	1·58 (0·85, 2·94)	n.a.	n.a.	11·43 (3·35, 6·53)
Namibia	3·39 (1·72, 5·56)	n.a.	n.a.	8·8 (2·67, 5·27)
Nicaragua	2·79 (1·46, 4·78)	n.a.	n.a.	9·02 (2·76, 5·43)
Niger	1·12 (0·59, 2·16)	0·4 (0·22, 0·83)	0·54 (0·28, 1·45)	5·63 (1·97, 3·72)
Nigeria	1·82 (0·94, 3·2)	n.a.	n.a.	9·4 (2·82, 5·55)
North Korea	0·93 (0·54, 2·01)	n.a.	n.a.	6·1 (2·14, 4)
Pakistan	1·29 (0·7, 2·48)	n.a.	n.a.	6·35 (2·08, 4·19)
Panama	11·37 (5·75, 17·62)	n.a.	n.a.	8·75 (2·58, 4·95)
Papua New Guinea	2·33 (1·24, 4·11)	0·82 (0·43, 1·46)	1·33 (0·68, 2·62)	10·36 (3·1, 6·06)
Peru	7·27 (3·7, 11·5)	n.a.	n.a.	9·56 (2·89, 5·68)
Rwanda	1·2 (0·63, 2·27)	n.a.	n.a.	5·32 (1·89, 3·59)
Senegal	1·38 (0·72, 2·55)	n.a.	n.a.	9·23 (2·78, 5·47)
Sierra Leone	1·17 (0·61, 2·23)	n.a.	n.a.	4·33 (1·62, 3·16)
Solomon Islands	3·01 (1·58, 5·13)	n.a.	n.a.	8·22 (2·56, 5·08)
Somalia	1·46 (0·78, 2·74)	0·59 (0·31, 1·11)	0·97 (0·5, 2·08)	7·02 (2·37, 4·37)
South Sudan	1·25 (0·65, 2·35)	n.a.	n.a.	6·45 (2·19, 4·08)
Sudan	2·84 (1·47, 4·81)	n.a.	n.a.	8 (2·64, 4·8)
Suriname	4·43 (2·28, 7·23)	n.a.	n.a.	9·51 (2·88, 5·66)
Sao Tome and Principe	2·93 (1·49, 4·87)	n.a.	n.a.	13·47 (3·83, 7·41)
Tanzania	1·48 (0·77, 2·7)	0·51 (0·27, 0·99)	0·64 (0·34, 1·59)	9·3 (2·79, 5·5)
Togo	1·23 (0·64, 2·32)	0·43 (0·23, 0·87)	0·55 (0·29, 1·43)	5·47 (1·93, 3·65)
Uganda	1·4 (0·73, 2·58)	n.a.	n.a.	6·05 (2·09, 3·91)
Vanuatu	4·46 (2·3, 7·31)	1·53 (0·78, 2·52)	2·41 (1·22, 4·19)	10·01 (3·01, 5·9)
Venezuela	7·38 (3·76, 11·66)	n.a.	n.a.	9·29 (2·82, 5·56)
Yemen	1·13 (0·62, 2·25)	n.a.	n.a.	2·2 (1·07, 2·26)
Zambia	1·39 (0·72, 2·56)	n.a.	n.a.	8·51 (2·59, 5·14)
Zimbabwe	2·58 (1·32, 4·35)	n.a.	n.a.	9·1 (2·74, 5·41)

n.a. = not applicable. Unit costs of deworming interventions have only been estimated in countries where the prevalence of hookworm and/or T. trichiura infection is > 20% and the prevalence of anaemia is > 40 % in line with World Health Organization recommendations. USD = United Stated dollars

Figure A2: Results from analysis of breakdown of unit costs between cost components averaged across WHO region.

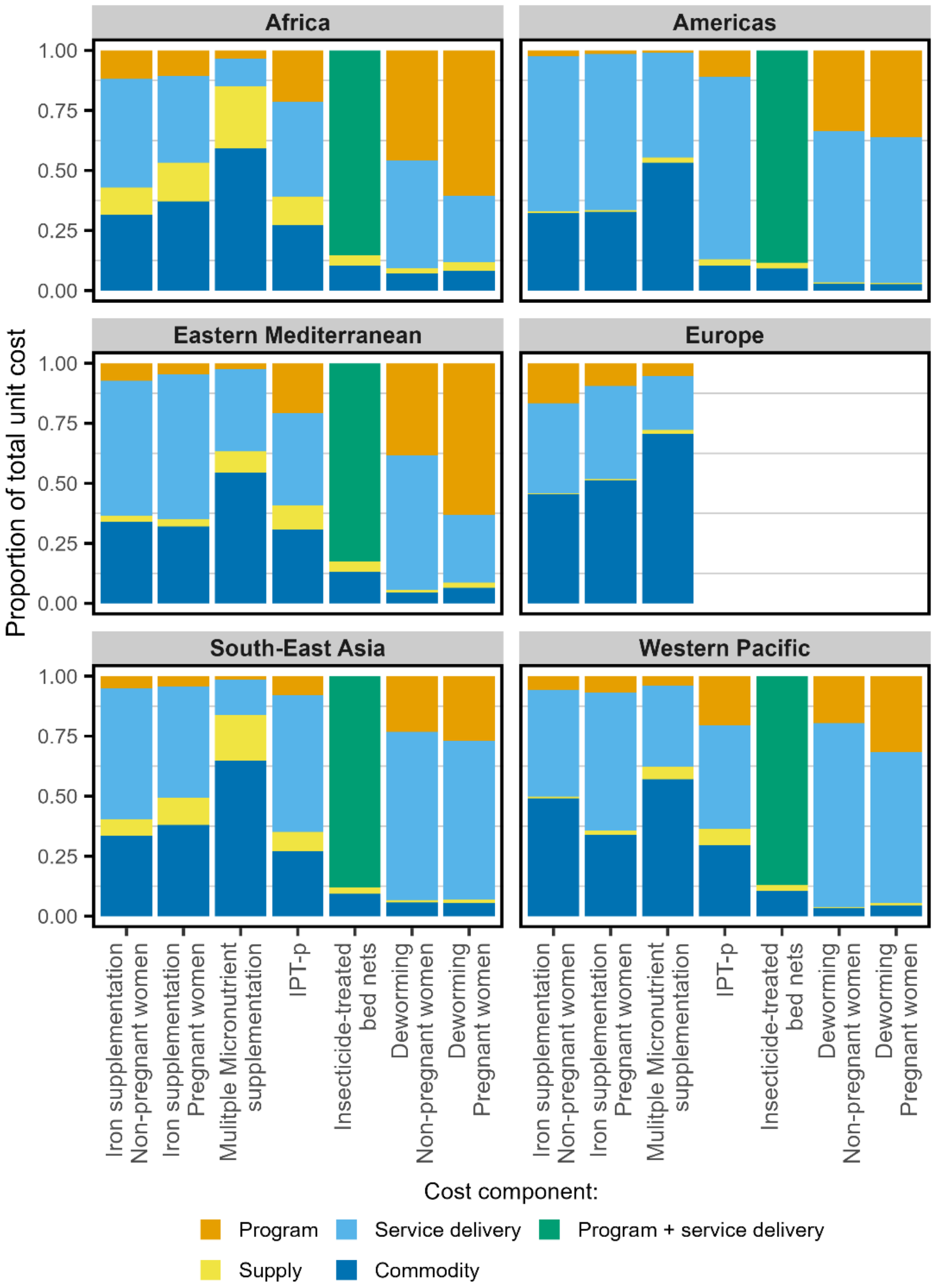

IPTp-SP = intermittent preventative treatment of malaria in pregnancy with sulfadoxine-pyrimethamine. Unit costs of deworming or malaria interventions are not shown for the European region as unit costs have not been estimated for interventions in settings where current WHO guidelines do not make explicit recommendations.

Figure A3: Results from the extreme scenario analysis exploring total uncertainty in overall unit costs averaged across WHO region. Upper and lower bounds shown here represent population-weighted means of the upper and lower estimates from all countries in each region.

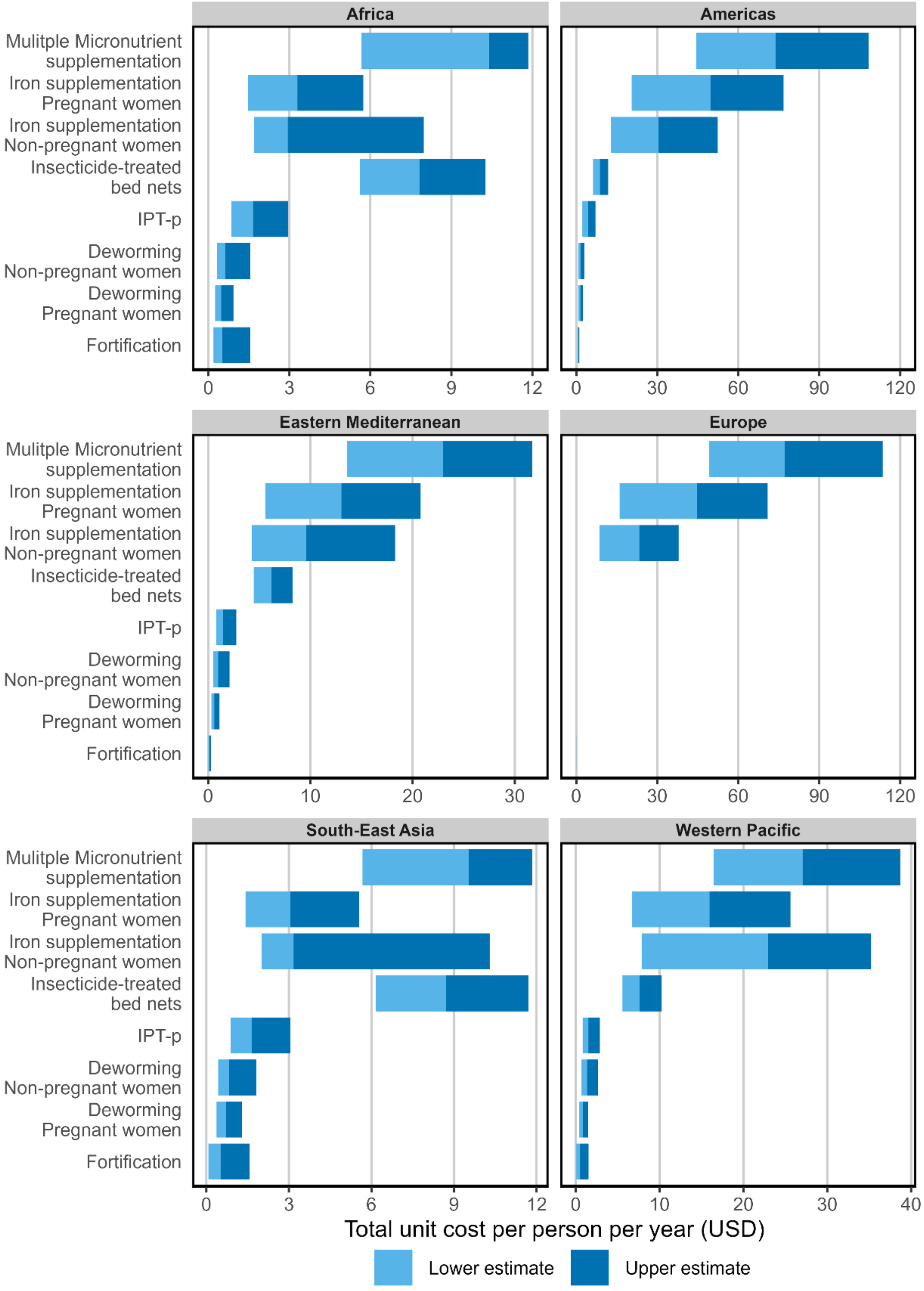

IPTp-SP = intermittent preventative treatment of malaria in pregnancy with sulfadoxine-pyrimethamine. Unit costs of deworming or malaria interventions are not shown for the European region as unit costs have not been estimated for interventions in settings where current WHO guidelines do not make explicit recommendations. Fortification costs are shown for the European region, but range is not visible at this scale.

3. References

1. World Health Organization. e-Library of Evidence for Nutrition Actions (eLENA). https://www.who.int/tools/elena/interventions#V (accessed 12 June 2024).
2. World Health Organization. Guideline: Intermittent iron and folic acid supplementation in menstruating women. Geneva, Switzerland: World Health Organization, 2011.
3. World Health Organization. Guideline: Daily iron supplementation in adult women and adolescent girls. Geneva, Switzerland: World Health Organization, 2016.
4. World Health Organization. WHO recommendations on antenatal care for a positive pregnancy experience. Geneva, Switzerland: World Health Organization, 2016.
5. World Health Organization. Guideline: fortification of rice with vitamins and minerals as a public health strategy. Geneva: World Health Organization, 2018.
6. World Health Organization. Guideline: fortification of wheat flour with vitamins and minerals as a public health strategy Geneva: World Health Organization, 2022.
7. World Health Organization. WHO guideline: fortification of maize flour and corn meal with vitamins and minerals. . Geneva: World Health Organization, 2016.
8. World Health Organization. WHO Guidelines for Malaria. Geneva, 2022.
9. World Health Organization. Preventive chemotherapy to control soil-transmitted helminth infections in at-risk population groups. Geneva Switzerland, 2017.
10. World Health Organization. WHO antenatal care recommendations for a positive pregnancy experience. Nutritional interventions update: Multiple micronutrient supplements during pregnancy. Geneva: World Health Organization, 2020.
11. Young N, Bowman A, Swedin K, et al. Cost-effectiveness of antenatal multiple micronutrients and balanced energy protein supplementation compared to iron and folic acid supplementation in India, Pakistan, Mali, and Tanzania: A dynamic microsimulation study. *PLoS Med* 2022; **19**(2): e1003902.
12. Kashi B, C MG, Kurzawa ZA, Verney AMJ, Busch-Hallen JF, De-Regil LM. Multiple Micronutrient Supplements Are More Cost-effective Than Iron and Folic Acid: Modeling Results from 3 High-Burden Asian Countries. *J Nutr* 2019; **149**(7): 1222-9.
13. Sanders GD, Neumann PJ, Basu A, et al. Recommendations for Conduct, Methodological Practices, and Reporting of Cost-effectiveness Analyses: Second Panel on Cost-Effectiveness in Health and Medicine. *JAMA* 2016; **316**(10): 1093-103.
14. Bertram MY, Lauer JA, Stenberg K, Edejer TTT. Methods for the Economic Evaluation of Health Care Interventions for Priority Setting in the Health System: An Update From WHO CHOICE. *Int J Health Policy Manag* 2021; **10**(11): 673-7.
15. Peña-Rosas JP, De-Regil LM, Dowswell T, Viteri FE. Daily oral iron supplementation during pregnancy. *Cochrane Database Syst Rev* 2012; **12**: Cd004736.
16. The World Bank. World Bank Country and Lending Groups. 2024.
17. Bahl K, Toro E, Qureshi C, Shaw P. Nutrition for a Better Tomorrow: Scaling Up Delivery of Micronutrient Powders for Infants and Young Children: Results for Development (R4D), 2013.
18. Lee KS, Kassab YW, Taha NA, Zainal ZA. A systematic review of pharmaceutical price mark-up practice and its implementation. *Explor Res Clin Soc Pharm* 2021; **2**: 100020.
19. Ahmad NS, Islahudin F. Affordability of essential medicine prices in Malaysia's private health sector. *Patient Prefer Adherence* 2018; **12**: 1231-7.
20. Roche ML, Samson KLI, Green TJ, Karakochuk CD, Martinez H. Perspective: Weekly Iron and Folic Acid Supplementation (WIFAS): A Critical Review and Rationale for Inclusion in the Essential Medicines List to Accelerate Anemia and Neural Tube Defects Reduction. *Adv Nutr* 2021; **12**(2): 334-42.
21. Stenberg K, Watts R, Bertram MY, et al. Cost-Effectiveness of Interventions to Improve Maternal, Newborn and Child Health Outcomes: A WHO-CHOICE Analysis for Eastern Sub-Saharan Africa and South-East Asia. *Int J Health Policy Manag* 2021; **10**(11): 706-23.
22. International Labour Organization. ILOSTAT.
23. Neogi SB, John D, Sharma J, et al. Cost-effectiveness of point-of-care devices for detection of anemia in community settings in India. *Clinical Epidemiology and Global Health* 2022; **14**: 100995.

24. Wisniewski J, Acosta A, Kolaczinski J, Koenker H, Yukich J. Systematic review and meta-analysis of the cost and cost-effectiveness of distributing insecticide-treated nets for the prevention of malaria. *Acta Trop* 2020; **202**: 105229.
25. Shekar M, Kakietek J, Dayton Eberwein J, Walters D. An Investment Framework for Nutrition: Reaching the Global Targets for Stunting, Anemia, Breastfeeding, and Wasting. Washington, DC: World Bank, 2017.
26. Shekar M, Kakietek J, Eberwein JD, Walters D. An Investment Framework for Nutrition: Reaching the Global Targets for Stunting, Anemia, Breastfeeding, and Wasting. Washington, DC: Directions in Development. World Bank 2017.
27. Baltussen R, Knai C, Sharan M. Iron Fortification and Iron Supplementation are Cost-Effective Interventions to Reduce Iron Deficiency in Four Subregions of the World. *The Journal of Nutrition* 2004; **134**(10): 2678-84.
28. Johns B, Baltussen R, Hutubessy R. Programme costs in the economic evaluation of health interventions. *Cost Effectiveness and Resource Allocation* 2003; **1**(1): 1.
29. Baltussen R, Knai C, Sharan M. Iron fortification and iron supplementation are cost-effective interventions to reduce iron deficiency in four subregions of the world. *J Nutr* 2004; **134**(10): 2678-84.
30. Fiedler JL, Macdonald B. A strategic approach to the unfinished fortification agenda: feasibility, costs, and cost-effectiveness analysis of fortification programs in 48 countries. *Food Nutr Bull* 2009; **30**(4): 283-316.
31. Alavi S, Bugusu B, Cramer G, et al. Rice Fortification in Developing Countries: A Critical Review of the Technical and Economic Feasibility. Washington, DC: United States Agency for International Development, 2008.
32. Turner HC, Lauer JA, Tran BX, Teerawattananon Y, Jit M. Adjusting for Inflation and Currency Changes Within Health Economic Studies. *Value Health* 2019; **22**(9): 1026-32.
33. Vassall A, S. Sweeney, Kahn J, et al. Reference Case for Estimating the Costs of Global Health Services and Interventions: Global Health Cost Consortium, 2017.
34. International Monetary Fund. Official exchange rate (LCU per US$, peroid average). International Financial Statistics: World Bank Group; 2023.
